# The TeMPO trial (treatment of meniscal tears in osteoarthritis): rationale and design features for a four arm randomized controlled clinical trial

**DOI:** 10.1186/s12891-018-2327-9

**Published:** 2018-12-01

**Authors:** James K. Sullivan, James J. Irrgang, Elena Losina, Clare Safran-Norton, Jamie Collins, Swastina Shrestha, Faith Selzer, Kim Bennell, Leslie Bisson, Angela T. Chen, Courtney K. Dawson, Alexandra B. Gil, Morgan H. Jones, Melissa A. Kluczynski, Kathleen Lafferty, Jeffrey Lange, Emma C. Lape, John Leddy, Aaron V. Mares, Kurt Spindler, Jennifer Turczyk, Jeffrey N. Katz

**Affiliations:** 1 Orthopaedic and Arthritis Center for Outcomes Research (OrACORe) and Policy and Innovation eValuation in Orthopedic Treatments (PIVOT), Department of Orthopaedic Surgery, Brigham and Women’s Hospital, 60 Fenwood Road, BTM 5016, Boston, MA 02115 USA; 2 Department of Physical Therapy, School of Health and Rehabilitation Sciences, University of Pittsburgh, Pittsburgh, PA USA; 3 Harvard Medical School, Boston, MA USA; 4 Section of Clinical Sciences, Division of Rheumatology, Immunology and Allergy, Brigham and Women’s Hospital, Boston, MA USA; 5 Departments of Epidemiology and Environmental Health, Harvard T. H. Chan School of Public Health, Boston, MA USA; 6 Department of Biostatistics, Boston University School of Public Health, Boston, MA USA; 7 Department of Rehabilitation Services – Physical and Occupational Therapy, Brigham and Women’s Hospital, Boston, MA USA; 8 Centre for Health, Exercise and Sports Medicine, School of Health Sciences, University of Melbourne, Parkville, Australia; 9 UBMD Department of Orthopaedics and Sports Medicine, Jacobs School of Medicine and Biomedical Sciences, State University of New York at Buffalo, Buffalo, NY USA; 10 Department of Orthopaedics, Brigham and Women’s Hospital, Boston, MA USA; 11 Department of Orthopaedic Surgery, Cleveland Clinic, Cleveland, OH USA; 12 Department of Orthopaedic Surgery, University of Pittsburgh Medical Center, Pittsburgh, PA USA

**Keywords:** Osteoarthritis, Meniscal tear, Randomized trial, Physical therapy, Placebo

## Abstract

**Background:**

Meniscal tears often accompany knee osteoarthritis, a disabling condition affecting 14 million individuals in the United States. While several randomized controlled trials have compared physical therapy to surgery for individuals with knee pain, meniscal tear, and osteoarthritic changes (determined via radiographs or magnetic resonance imaging), no trial has evaluated the efficacy of physical therapy alone in these subjects.

**Methods:**

The Treatment of Meniscal Tear in Osteoarthritis (TeMPO) Trial is a four-arm multi-center randomized controlled clinical trial designed to establish the comparative efficacy of two in-clinic physical therapy interventions (one focused on strengthening and one containing placebo) and two protocolized home exercise programs.

**Discussion:**

The goal of this paper is to present the rationale behind TeMPO and describe the study design and implementation strategies, focusing on methodologic and clinical challenges.

**Trial registration:**

The TeMPO Trial was first registered at clinicaltrials.gov with registration No. NCT03059004. on February 14, 2017.

**Electronic supplementary material:**

The online version of this article (10.1186/s12891-018-2327-9) contains supplementary material, which is available to authorized users.

## Background

### Introduction

Symptomatic radiographic knee osteoarthritis (OA) is a costly and disabling condition that affects over 14 million people in the United States [1]. Meniscal tears are identified on magnetic resonance imaging (MRI) in 60–90% of persons with symptomatic knee OA [2, 3]. While these tears can be clinically silent, symptomatic meniscal tear is diagnosed by clinicians based primarily on patient-reported symptoms of knee pain, often accompanied by mechanical manifestations [2–6]. Currently, arthroscopic partial meniscectomy (APM) with physical therapy (PT) or PT alone are frequently recommended treatments for adults with knee pain, meniscal tear, and evidence of OA [7]. Several recent randomized controlled trials (RCTs) have reported that subjects randomized to APM (with or without PT) reported similar pain and functional outcomes one year after surgery compared to those randomized to PT only [8–14]. These trials suggest that PT alone is a reasonable initial treatment in persons with symptomatic degenerative meniscal tear.

Since no trials have examined the efficacy of PT as compared with a control group or placebo for conservative management of symptomatic meniscal tear, [15, 16] we do not know whether the pain relief and improved knee function associated with PT-based regimens are attributable to physiologic effects of exercise and strengthening or whether the attention provided by the physical therapist (placebo) is responsible, at least in part, for some of the therapeutic benefit. A review of trials for diverse chronic pain conditions that included both placebo and ‘no treatment’ arms concluded that the placebo arms improved, on average, by an effect size of 0.32 standard deviations (SD) over no treatment, [17] providing further evidence of the importance of studying the effect of placebo in PT-based treatment for symptomatic meniscal tear.

Several placebo-controlled trials have been carried out in subjects with knee OA, a population with average pain levels similar to persons with symptomatic meniscal tear and knee OA [18–25]. In most of these trials, moderate improvements in pain and function were reported for both active PT and placebo arms, indicating that placebo (such as sham ultrasound or sham taping) effects play an important role in the symptom relief provided by PT-based treatments. In addition to comparing active PT with placebo, our trial considers whether face-to-face interactions with a physical therapist coupled with a rigorous home exercise program can provide greater pain relief than the home exercise program alone.

We designed a four-arm multicenter randomized controlled trial to establish the efficacy of a typical PT regimen compared to placebo and home exercise alone for individuals aged 45 to 85 with symptomatic meniscal tear and osteoarthritic structural changes undergoing non-operative management. The four arms include: 1) a protocolized home exercise program (pamphlet, video directions); 2) a protocolized home exercise program with adherence optimization (text or email messages); 3) a protocolized home exercise program with adherence optimization plus therapist-directed placebo PT; and 4) a protocolized home exercise program with adherence optimization plus therapist-directed exercise and manual therapy. The primary outcome for the trial is the 3-month change in the Knee Injury and Osteoarthritis Outcome Score (KOOS) pain score.

### Hypotheses

The hypotheses include:

1. Arm 4 (protocolized home program, adherence optimization, in-clinic physical therapist-delivered exercise and manual therapy) is more efficacious than Arm 1 (protocolized home program alone). This analysis contrasts the putatively most potent intervention against the least potent.
2. Arm 4 (protocolized home program, adherence optimization, in-clinic physical therapist-delivered exercise and manual therapy) is more efficacious than Arm 2 (protocolized home program with adherence optimization). This contrast isolates the effect of the in-clinic physical therapist-delivered intervention of exercise and manual therapy.
3. Arm 4 (protocolized home program, adherence optimization, in-clinic physical therapist-delivered exercise and manual therapy) is more efficacious than Arm 3 (protocolized home program with adherence optimization and placebo PT). This contrast compares the effects of “active” PT and placebo PT, both given along with protocolized home exercises.
4. Arm 3 (protocolized home program, adherence optimization, in-clinic placebo PT) is more efficacious than Arm 2 (protocolized home program with adherence optimization). This contrast isolates the effect of attending an in-clinic program that provides face to face contact and support by clinicians with essentially no physiological effect directed at musculoskeletal impairments.
5. Arm 2 (protocolized home program with adherence optimization) is more efficacious than Arm 1 (protocolized home program without adherence optimization). This contrast isolates the effect of the adherence optimization program.

## Methods

The methods section of this paper is organized into two broad categories: Study Design and Trial Implementation. Study Design reviews the epidemiological, statistical, and trial design components considered when planning the TeMPO trial. Trial Implementation reviews highlights of the trial’s execution.

### Study design

#### Trial design and structure

TeMPO is a multi-center, four-arm, parallel 1:1:1:1 assessor-blinded RCT. The four clinical sites are: Brigham and Women’s Hospital (BWH), Boston, Massachusetts; University at Buffalo Jacobs School of Medicine and Biomedical Sciences (UB-SMBS), Buffalo, New York; The Cleveland Clinic (CC), Cleveland, Ohio; and The University of Pittsburgh Medical Center (UPMC), Pittsburgh, Pennsylvania. This study is registered at Clinicaltrials.gov (NCT03059004) and was approved by a Single Institutional Review Board (sIRB) hosted at Brigham and Women’s Hospital covering all clinical sites. This paper describes the TeMPO trial in accordance with the Standard Protocol Items: Recommendations for Interventional Trials (SPIRIT) guidelines [26].

#### Patient sample

We plan to recruit approximately 214 subjects per arm, 856 total. Recruitment started in February 2018 and will likely run through summer 2022.

Table 1 details the inclusion and exclusion criteria for the TeMPO trial. Eligible subjects must meet each inclusion criterion and not meet any exclusion criterion. All subjects must be 45–85 years old seeking treatment for knee pain. The enrolling physcians must affirm that the pain is due in part to a meniscal tear. Subjects must also have imaging evidence of meniscal tear and mild to moderate osteoarthritic changes. Because the majority of meniscal tears identified in middle-aged and older persons are asymptomatic, [2, 3, 27, 28] TeMPO inclusion criteria stipulate that the location (medial vs. lateral) of reported meniscal tear-like knee symptoms must concur with the location (medial vs. lateral compartment) of the MRI-documented meniscal tear. The study defines ‘osteoarthritis change’ as evidence of partial or full thickness cartilage lesions on MRI in any knee compartment. We opted for MRI evidence of OA because of the general recognition that the OA process begins before being detectable on plain radiographs [29].

**Table 1:** TeMPO Trial Inclusion and Exclusion Criteria

Inclusion Criteria	
Age 45–85	
Lives within 60 miles of a clinical site	
English speaking	
Has access to email	
Not living in a nursing home	
Physician suspects meniscal tear with symptoms	
WOMAC* (knee) pain ≥15	
Meniscal tear present on MRI^†^ (taken in the last year) and aligning with symptomatic compartment	
Degenerative cartilage changes on MRI (taken in the last year)	
Exclusion Criteria	
Equal bilateral symptoms of meniscal tear (in both knees)	
Deemed by physician as “not fit” for a trial of conservative knee treatments due to behavioral or social reasons, being seen for reason other than knee pain, indication for surgery, or contraindication for physical therapy	
Pregnant	
Dementia documented in medical record	
Inflammatory arthritis or avascular necrosis of the knee	
Surgical exclusion (surgery within 6 months on index knee, planned TKR‡ within 6 months, prior knee replacement, or meniscal surgery on index knee)	
Claiming worker’s compensation for knee pain	
Requires use of wheelchair or walker	
Contraindication to or concerning findings on MRI	
Exercise exclusion: physician recommends against unsupervised exercise	
Daily use of strong opioids	
2 deferments due to injection or lack of availability	
Kellgren Lawrence grade 4 osteoarthritis on X-ray	
Currently in a knee physical therapy regimen for 2 weeks or more at a frequency of once a week or more	
Knee pain arising from trauma < 21 days ago	
Physician exclusion (opted out, unfit for trial participation)	

* Western Ontario and McMaster Universities Osteoarthritis Index

† Magnetic resonance imaging

‡ Total knee replacement

Subjects with pain due to recent (≤ 21 days) traumatic injury are excluded, as we did not wish to enroll subjects whose symptoms were self-limited. Subjects with prior history of APM or joint replacement in the index knee, any other surgery on the index knee within the past 6 months, inflammatory arthritis, or advanced OA (Kellgren-Lawrence [KL] 4 on x-ray) are also excluded. Any subject who receives an injection in their index knee must wait at least 4 weeks prior to randomization because injections can temporarily lower baseline pain levels, thereby masking pain and/or functional impairments.

#### Randomization and blinding

Subjects who meet all eligibility criteria are randomized to one of the four treatment arms in a 1:1:1:1 ratio, in randomly varying blocks of size 4 and 8, stratified by KL-grade (0–2 or 3) and by clinic site. The research coordinator (RC) at each site randomizes each subject in real time using the secure, online randomization module within the TeMPO REDCap (Research Electronic Data Capture) data capture system hosted by Partners HealthCare. Study physicians, musculoskeletal (MSK) assessors, and any other research personnel involved in the collection of the primary outcome are blinded to subject arm assignment. Interventions

**Fig. 1:**
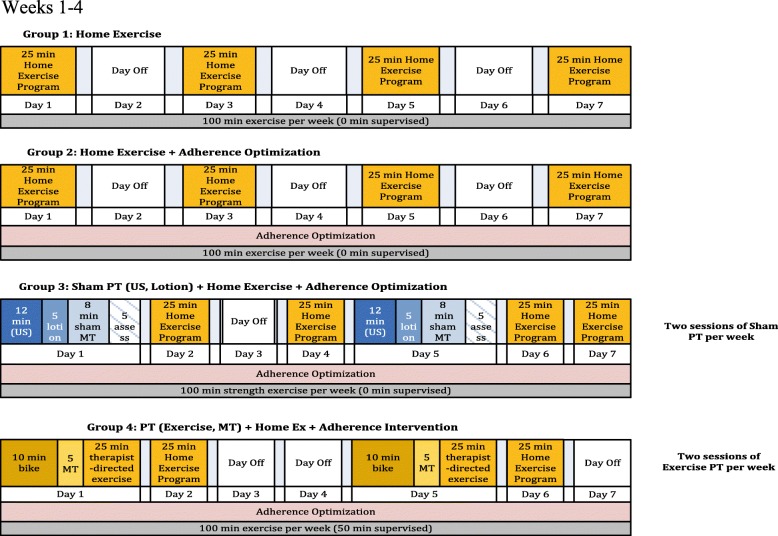
Exercise Dose Diagrams in the TeMPO Trial. Exercise dose diagram for weeks 1–4 of trial intervention. All subjects complete 100 min of a strengthening based home program (Arms 1–3) or a combination of the home program and therapist-directed manual therapy and exercise per week (Arm 4) per week. Subjects in Arms 2, 3, and 4 receive adherence optimization consisting of motivational adherence support text messages three times a week and a health information pamphlet once every other week. Subjects in Arm 3 receive a placebo physical therapy intervention consisting of placebo ultrasound (US), placebo lotion application, placebo manual therapy (MT), and placebo assessment twice a week. Subjects in Arm 4 receive a true physical therapy intervention consisting of manual therapy (MT) and supervised strengthening/functional exercise two times a week and complete the protocolized home exercise program two times a week. Subjects are not instructed to exercise in any particular order or sequence, only at the designated frequency

**Fig. 2:**
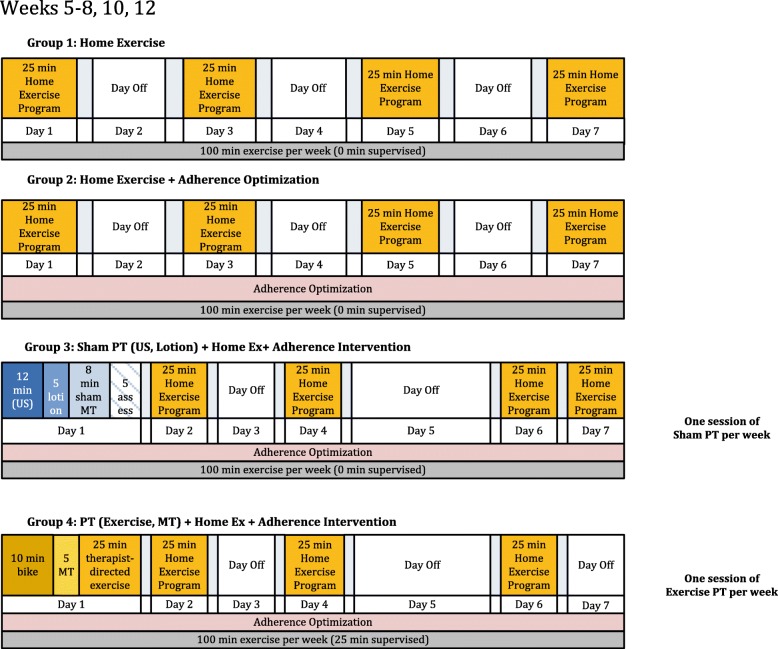
Exercise dose diagram for weeks 5–8, 10, 12 of trial intervention. All subjects complete 100 min of a strengthening based home program (Arms 1–3) or a combination of the home program and therapist-directed manual therapy and exercise per week (Arm 4) per week. Subjects in Arms 2, 3, and 4 receive adherence optimization consisting of motivational adherence support text messages three times a week and a health information pamphlet once every other week. Subjects in Arm 3 receive a placebo physical therapy intervention consisting of placebo ultrasound (US), placebo lotion application, placebo manual therapy (MT), and placebo assessment twice a week. Subjects in Arm 4 receive a true physical therapy intervention consisting of manual therapy (MT) and supervised strengthening exercise once a week and complete the protocolized home exercise program three times a week. Subjects are not instructed to exercise in any particular order or sequence, only at the designated frequency

**Fig. 3:**
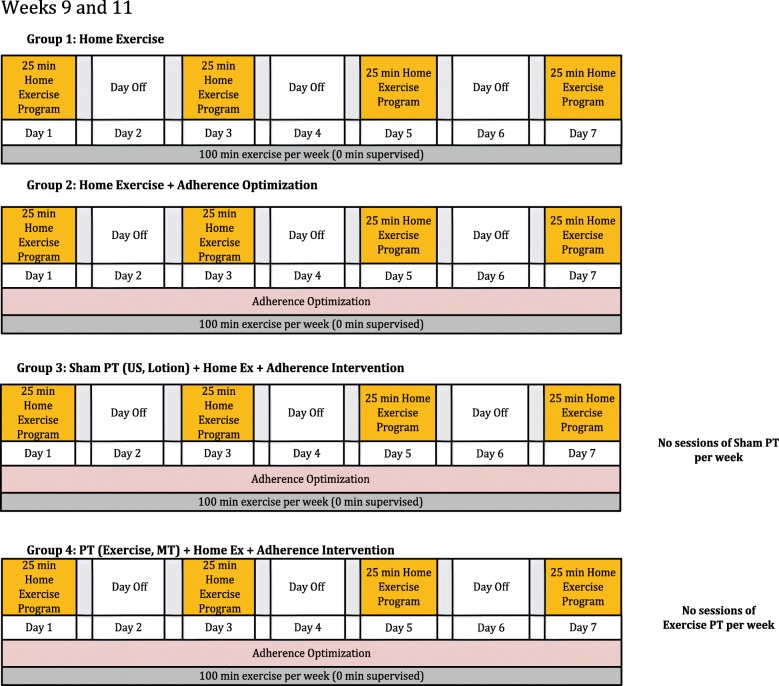
Exercise dose diagram for weeks 9 and 11 of trial intervention. All subjects complete 100 min per week of a strengthening based home program at home. Subjects in Arms 2, 3, and 4 receive adherence optimization consisting of motivational adherence support text messages three times a week and a health information pamphlet once every other week. Subjects in Arms 3 and 4 do not go to clinic for additional treatment in these weeks. Subjects are not instructed to exercise in any particular order or sequence, only at the designated frequency

**Fig. 4:**
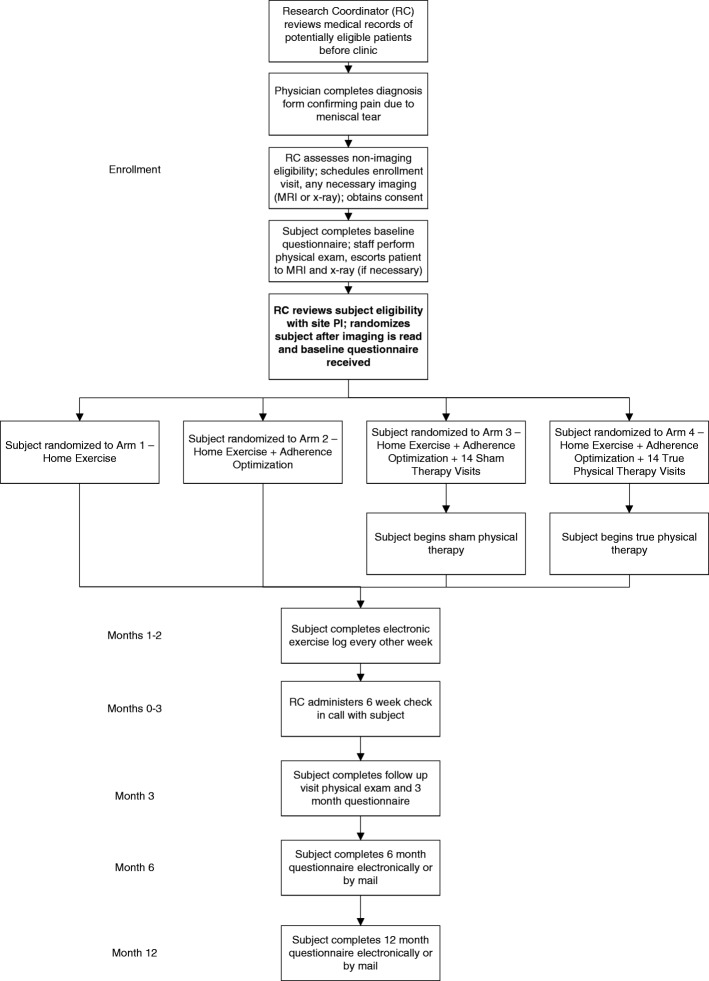
Flow diagram depicting enrollment and follow-up procedures in the TeMPO trial

The four TeMPO intervention arms are depicted in Table 2 and described below according to the Template for Intervention Description and Replication (TiDIER) guidelines [30]. Each intervention is balanced such that all subjects receive 100 min of a strengthening-focused exercise program per week (Figs. 1, 2 and 3). The intervention period for each arm lasts for 12 weeks. Specific timelines and procedures for trial enrollment, trial arm activities, and follow-up schedules are included in the flow diagram in Fig. 4.

**Table 2:** Overview of the different intervention components

Arm	Protocolized Home Exercise Program	Adherence Optimization	Placebo Therapy (14 sessions)	Supervised Exercise & Manual Therapy (14 sessions)
Arm 1	**✓**			
Arm 2	**✓**	**✓**		
Arm 3	**✓**	**✓**	**✓**	
Arm 4	**✓**	**✓**		**✓**

##### Protocolized home exercise program (arms 1–4)

All subjects receive identical standardized, progressive, self-guided protocolized home exercise programs consisting of stretches (quadriceps and hamstrings); progressive strengthening exercises targeting hamstrings, quadriceps, gluteus medius, and gluteus maximus muscle groups; and functional/neuromuscular exercises (Table 3). Randomized subjects receive an instructional pamphlet and video, which is available both on the web and on a flash drive. Participants are also given adjustable ankle weights (adjustable in 1-lb increments from 0 to 10 lbs) and detailed guidelines for progression of weights. The subjects receive no direct instruction from a physical therapist for this standardized, protocol-driven home exercise program. Subjects are instructed to progress in the program when the exercises become easier as long as there is only minimal knee pain or soreness 24 h after exercising. Alternate positioning or exercises are suggested for most program components for subjects who are unable to perform the primary form of the exercise or stretch. The instructional pamphlet and a link to the home exercise video given to subjects are included in Additional file 1.

**Table 3:** Protocolized home exercise program exercises and progression

Targeted Muscles^a^	Initial	Intermediate	Advanced
Stretches	Hamstrings 2x30s^b^ Quadriceps 2x30s	Hamstrings 2x30s Quadriceps 2x30s	Hamstrings 2x30s Quadriceps 2x30s
Gluteus Maximus	Bent over hip extension with knee bent without weight; OR *Bridging*	Bent over hip extension with knee bent with weight (1–5 lbs)	Bent over hip extension with knee bent with weight (6–10 lbs)
Gluteus Medius	Side-lying straight leg lift without weight; OR *Clamshell*	Side-lying straight leg lift with weight (1–5 lbs)	Side-lying straight leg lift with weight (6–10 lbs)
Quadriceps	Straight leg raise without weight; OR *Seated knee extension without weight*	Straight leg raise with weight (1–5 lbs); OR *Seated knee extension with weight (1–5 lbs)*	Straight leg raise with weight (6–10 lbs); OR *Seated knee extension with weight (6–10 lbs)*
Hamstrings	Standing knee bent without weight	Standing knee bent with weight (1–5 lbs)	Standing knee bent with weight (6–10 lbs)
Functional	Mini wall squats	Regular chair squat	Staggered leg chair squat

^a^All exercises, except where indicated were done in 3 sets of 12 reps 4 times per week. Subjects were encouraged to begin at as low as 3 sets of 8 reps and work their way to 12 repetitions per set as tolerated

^b^2x30s refers to two reps of a 30 s hold of each stretch

##### Adherence Optimimization (arms 2–4)

Subjects randomized to Arms 2–4 also receive ‘adherence optimization’ in the form of short message service (SMS) text messages three times a week as well as 6 different health information pamphlets mailed throughout the active intervention. The SMS messages are anchored in behavioral theories (Theory of Planned Behavior, [31, 32] Theory of Reasoned Action, [32] Social Cognitive Theory, [33] Information Motivation on Behavioral Skills Model [34]) and developed to encourage behavior change. Message content was based on previous studies utilizing SMS adherence support messages with a particular focus on barriers and facilitators to exercise identified in people with OA [35–47]. The health pamphlets provide information about the relevant knee anatomy, the biomechanical rationale for select exercises included in the protocolized home exercise program, healthful recipes, strategies for addressing chronic pain, and motivational quotes. The texts and pamphlets are designed to increase participants’ engagement in the study through consistent exposure to informative materials and to address barriers to exercising at home. Subjects in these arms also sign an Adherence Contract at the beginning of the intervention period attesting that they will perform the exercises to the best of their ability.

##### PT placebo (arm 3)

In addition to the home exercise program, subjects in Arm 3 receive a maximum of 14 half-hour placebo PT sessions in clinic with a trained and licensed physical therapist over the 3-month period (Fig. 1). While the total duration of in-person contact time with therapists in Arms 3 and 4 is equivalent, subjects in Arm 3 receive no active treatments that have a known physiologic effect on knee pain and functional impairments. Also, physical therapists do not provide any instructions for the home exercise program. The placebo PT regimen consists of four components: 1) an assessment of current knee symptoms (5 min); 2) placebo ultrasound (intensity of the ultrasound is set to 0; 14 min); 3) placebo lotion application applied with no force along the mid-thigh and tibial area near the ankle (4–5 min); and 4) placebo manual therapy, in which the therapist applies minimal force to non-articular areas on the medial and lateral aspects of the knee (e.g. femoral condyles), with no joint mobilization, and guides hip internal/external rotation or instructs subjects to invert/evert or dorsi/plantar flex the ankle (7–8 min; Additional file 2).

##### Active physical therapy (arm 4)

Subjects in Arm 4 receive an “active” physical therapy regimen in clinic with a licensed physical therapist of the same duration and frequency as treatment for subjects in Arm 3 (Fig. 1). Each session consists of: 1) manual therapy consisting of soft tissue mobilization, joint mobilization, and manual stretching of tissues around the knee (5 min); and 2) therapist-directed strengthening and functional exercises, targeting the gluteus maximus, gluteus medius, hamstrings, and quadriceps muscles (25 min). Therapists can alter the relative proportions of manual therapy, strengthening, and stretching exercises if clinically indicated. Therapists also increase or decrease the difficulty of the exercises as the subject’s pain and progress warrant. Specific exercises and manual therapy procedures are selected from a menu of possible exercises and procedures designed by the TeMPO physical therapists prior to the beginning of the trial (Additional file 3). Choosing in this way from finite options that target the same muscle groups provides therapists with flexibility to tailor treatments to subjects (as per usual clinical practice) while ensuring consistency in approach across trial therapists and clinical sites.

The in-clinic program and the protocolized home exercise program were developed after a review of the literature and consultation with an expert panel of 10 physical therapists from around the United States. We designed the program to meet American College of Sports Medicine (ACSM) and American Physical Therapist Association (APTA) guidelines [16]. The details of both programs will be reported elsewhere (Safran-Norton and colleagues, in preparation).

#### Data collection, analytic procedures, and statistical considerations

##### Overview of follow-up procedures

Outcome measures are assessed via questionnaires and a physical examination of the knee, including performance-based tests (Table 4). Study questionnaires are administered at baseline and at 3, 6 and 12 months post-randomization, and the brief MSK exam is performed at baseline and at 3 months by a blinded, study-certified assessor. Performance-based tests for the MSK exam are carried out according to Osteoarthritis Research Society International (OARSI) recommendations [48]. Study staff making reminder telephone calls to subjects relating to questionnaires and performing data entry are blinded to subject treatment assignment. Subjects are also asked to complete short home exercise logs online every other week during the intervention period (6 total logs) to monitor knee-related pain and adherence to the protocolized home exercise regimen.

**Table 4:** Outcomes assessed by questionnaire and musculoskeletal (MSK) examination

Form of Assessment	Outcome Measure	Reference
Questionnaire (baseline, 3-, 6-, and 12-months)	KOOS^a^ Knee Survey: Pain	[66]
	KOOS Knee Survey: Symptoms	[66]
	KOOS Knee Survey: Function, sports and recreational activities	[66]
	KOOS Knee Survey: Function, daily living	[66]
	KOOS Knee Survey: Quality of Life	[66]
	The Neuropathic Pain Scale	[67]
	The Widespread Pain Index	[68]
	The Mental Health Inventory – 5 (MHI-5)	[69]
	The Self-Administered Comorbidity Questionnaire	[70]
	EQ-5D	[71]
	The Pain Catastrophizing Scale	[72]
	Medical Resource Utilization	N/A
	Work Productivity and Activity Impairment Questionnaire: Specific Health Problem V2.0	[73]
MSK^b^ Exam (baseline and 3-months)	Quadriceps, Hamstrings, and Gluteus Medius Strength	N/A
	30 s sit-to-stand	[48]
	40 m fast-paced walk	[48]
	Hamstring, gastroc, and quadricep muscle length	N/A
	Knee range of motion	N/A
	Single leg balance test	N/A

^a^KOOS = Knee Osteoarthritis Outcome Score

^b^MSK = Musculoskeletal

##### Statistical considerations

*Primary Outcome:* The primary outcome is the difference in KOOS Pain scores between baseline and 3 months of follow-up. The primary analysis will follow an intention-to-treat approach. We will first use a four-level categorical treatment variable to test for an overall effect on KOOS pain and then examine the five contrasts specified in the hypotheses in separate models, adjusting for any baseline features that are imbalanced despite randomization.

*Secondary Outcomes:* Proposed secondary outcomes include 3-month change in KOOS function and EQ-5D quality of life measures, and 3-month change in the battery of tests performed during the MSK examination (Table 4). In exploratory analyses we will adjust for changes in the strength variables to evaluate whether changes in KOOS pain from baseline to 3 months are explained by changes in quadriceps, hamstring, or gluteus medius strength. An additional secondary outcome is the durability of the pain decrement associated with each treatment arm. We will evaluate treatment durability among those subjects with clinically meaningful improvement in KOOS (8–10 points [49, 50]) in the first 3 months. Among these subjects, we will assess the proportion across treatment arms maintaining at least an 8-point improvement at the 12-month follow-up.

*Binary Outcomes:* We recognize that subjects who receive a total knee replacement (TKR), APM, or injection during the course of trial intervention and follow-up may report improvements or exacerbations in pain that are attributable to these additional interventions rather than to the treatment they were randomized to receive. To address this issue, we will also perform analyses using a binary ‘failure’ outcome in which ‘failure’ is defined as undergoing TKR, APM, or injection or failing to improve in KOOS pain by 8 points in the first 3 months of the trial. We will calculate the proportion of subjects in each arm that fail using this binary outcome.

*Bonferroni Correction:* Given that three of the pairwise comparisons could influence clinical practice (Arms 4 v. 1; Arms 4 v. 2; Arms 2 v. 1), we will apply a Bonferroni correction with a critical *p*-value of 0.0167 (0.05/3). We do not consider comparisons involving Arm 3 in the Bonferroni correction because the placebo arm is not used in clinical practice.

*Power and sample size:* While we hypothesize a clinically meaningful difference of 8–10 points for the comparison of Arm 4 vs. Arm 1, we anticipate smaller differences for the other comparisons and have powered the study to detect such differences. Thus, TeMPO was powered to detect a difference of 0.33 SD on the KOOS pain scale. As our prior studies in this population reported a KOOS pain scale SD of 16 points at baseline, [8] we are powered to detect a 5.3 point difference on the KOOS Pain scale (16 × 0.33). We also accounted for an anticipated dropout rate of 10%.

We will address dropout and other forms of missing data by recording the reasons for dropout, specifically considering our binary treatment failure variables (APM, TKR, injection). We will determine whether the frequency of dropout differs across study arm with a primary assumption that data will be missing at random and dropout will not depend on unobserved outcomes. We will examine alternative assumptions in sensitivity analyses using a multiple imputation approach to impute a range of plausible values for missing outcomes [51].

*Cost effectiveness analysis:* We will perform a cost effectiveness analysis using data from the TeMPO Trial to investigate the relationship between costs and quality-adjusted life years (QALYs) gained for each intervention. We will perform the analysis with the Osteoarthritis Policy (OAPol) Model, a widely published and validated computer simulation model of the natural history, costs, and outcomes of care for persons with knee OA [52–57]. The analysis will adhere to the guidelines put forth by the 2016 Second Panel on Cost Effectiveness in Health and Medicine [58]. Input data on the costs and effectiveness of the intervention will be obtained from the TeMPO Trial as well as published sources. QALYs in each arm will be estimated using the Euroqol EQ5D instrument, administered in the baseline and 3-month follow-up TeMPO questionnaires. The analysis will be performed both from the provider and the societal perspectives and will assume 3% annual discounting of costs and QALYs, per panel recommendations.

### Trial implementation

#### Data management

Study data will be collected and managed using REDCap electronic data capture tools hosted by Partners HealthCare Research Computing, Enterprise Research Intrastructure and Services group. REDCap is a secure, web-based application designed to support data capture for research studies [59]. The study staff at BWH oversee the data management for all study data and is responsible for data collection and entry for all the 6- and 12-month questionnaires regardless of study site. All other assessments, including the baseline and 3-month questionnaires, are handled directly by the clinical sites.

#### Staff training

We developed comprehensive manuals of operating procedures for data entry, recruitment and retention, MSK assessments, and PT treatment protocols. Training for all site research coordinators was centralized at the BWH data coordinating site. Research coordinators reviewed manuals of operations for trial procedures and instructional videos on how to use the REDCap data management system. Coordinators participated in a series of conference calls and one in-person meeting with the project manager, data manager, and principal investigator at BWH to review protocols and address feasibility at their respective clinical sites. Lastly, coordinators completed 8 h of hypothetical recruitment and randomization scenarios, which evaluated their understanding of both trial and data entry protocols.

Therapists and MSK assessors were trained to ensure that all trained staff received the same instruction. We developed detailed written protocols and live video presentations of the MSK exam and Arms 3 and 4 physical therapy administration. Each site identified a designated PT and MSK exam certifier who used these materials to train and certify staff members through a series of in-person training sessions. All sites were sent the same assessment materials (straps, dynamometers, goniometers, etc.) in order to standardize the MSK assessment across study centers.

#### Equipoise and placebo intervention

As TeMPO contains a placebo physical therapy arm, therapist equipoise – impartiality between the ‘true’ and placebo treatment arms – is essential. Early in the trial design process, we assessed equipoise among interested therapists who might deliver the interventions. Investigators clarified that therapists who were uncomfortable with providing placebo interventions should not participate in the trial. The same therapists deliver both the placebo and ‘true’ PT regimens to eliminate bias in treatment effect by the personal qualities of individual therapists. Once therapists were selected, discussions were held with all therapists to determine the specific components of the placebo and true PT interventions. PT investigators considered feasibility and plausibility of the placebo components and reviewed similar placebo PT interventions used in other trials. During trial operation, regular therapist check-ins are held each month to discuss any concerns in the PT treatment arms. As subjects are informed of placebo elements in the trial but blinded to the placebo itself, we will assess the success of subject blinding in the 12-month questionnaire.

#### Recruitment

*Pre-screening:* During the ongoing trial recruitment phase, RCs at all sites conduct a brief medical record review of all patients scheduled to see participating clinicians to search for TeMPO exclusion criteria (Table 1). Reviews are conducted on all patients meeting three basic criteria: 1) age of 45–85 years, 2) presenting with knee pain, and 3) not presenting for a pre- or postoperative visit.

*Screening by Clinician:* For patients who pass pre-screening, clinicians complete a physician diagnosis form on which they document radiograph and MRI findings and rate the likelihood that the subject’s pain arises, at least in part, from meniscal tear after seeing the patient. When a patient’s symptoms are rated as probably or definitely due to meniscal tear and the patient appears to meet all other inclusion criteria, the clinician introduces the study to patient.

*Screening by RC:* RCs meet with those who are interested in TeMPO to assess all remaining eligibility criteria, provide an in-depth explanation of the trial, and enroll interested and eligibile patients. For instances in which the RC is unable to meet in person with interested potential subjects, we have developed telephone protocols to maximize recruitment.

#### Enrollment visit

Once a potential participant agrees to enroll, subjects undergo a baseline visit comprised of 1) providing written consent; 2) baseline questionnaire completion; and 3) MSK assessment. Any required imaging will also be obtained at the time of the enrollment visit.

#### Imaging

Knee MRIs and standing bilateral x-rays must both be obtained within one year of the clinic date to be valid for use in determining study eligibility. Potential subjects are scheduled for imaging when no images exist, images are older than the one year, or if the x-ray is non-weightbearing. A qualifying meniscal tear for the TeMPO study is defined as a signal abnormality on MRI extending to the surface of the meniscus on at least one slice in the same compartment (lateral vs. medial) as the one identified by the enrolling physician as responsible for the symptoms. In addition to meniscal tear, eligible subjects must have at least one cartilage defect or fissure, of any depth and any area, on any joint surface. Each center uses a basic set of MRI sequences consisting of Axial T2 FS, Sagittal PD, Sagittal PD FS, and Coronal PD, which are read by a study radiologist.

#### Post-randomization procedures

Immediately after randomization, coordinators call subjects informing them of their treatment assignment, mail a letter and package including all materials necessary for their treatment (e.g. home exercise instructions and weights), and email a copy of the randomization assignment letter to the subjects. Subjects in arms with adherence optimization receive pre-written text messages via a Python program powered by the Twilio REST API and developed by the study team.

#### Intervention Fidelity

To maintain study staff fidelity to the intervention, rigorous, annual recertification trainings are performed. Furthermore, to prevent drift in the standardization of the PT interventions, therapists document detailed reports in REDCap after each PT visit in addition to standard required clinic documentation. They record the intervention components completed and the amount of time time spent on each. These records are reviewed by study staff and therapists on monthly conference calls.

#### Optimizing engagement

Coordinators call subjects to explain treatment assignments at the time of randomization and then follow-up at 6 weeks by telephone to check in, answer questions, and remind subjects to complete the different intervention components and assessments. Mailings, pamphlets, and introductory materials were designed to be engaging, easy to understand, and informative. For subjects randomized to receive in-clinic treatments, the study covers the cost of PT visits and provides free parking to reduce barriers to attending therapy sessions. Monetary rewards are provided to subjects who complete questionnaires (15 USD per questionnaire) and the home exercise logs (20 USD if at least 5 of 6 logs are completed as well as the opportunity to win a monthly drawing with a monetary reward determined by log completions) [60–62].

#### Follow-up

After completing the 12-week intervention, subjects return to the clinical center for an in-person follow-up assessment with the same assessor that performed the baseline MSK examination. Subjects can complete the questionnaire during the in-person visit if they have not done so already to optimize response rate. Questionnaires are sent either in hard copy or electronically, based on subject preference, at the 6- and 12-month time-points with reminder calls administered by study staff to minimize loss to follow-up.

#### Adverse event and protocol deviations reporting

Adverse events (AE) are defined as untoward medical incidents occurring during the conduct of a research study, which may or may not have a causal relationship with the study procedures [63]. AEs are regarded as serious or non-serious. Serious adverse events (SAE) in TeMPO include: 1) death from any cause; 2) an overnight stay in the hospital as a result of medical problems or emergent surgery; and 3) a total knee replacement or arthroscopic partial meniscectomy surgery. TeMPO also tracks three classes of non-serious adverse events: 1) emergency room visits for any reason; 2) an exacerbation of knee pain that either precludes walking or requires the subject to rely upon an assistive device such as a cane or crutch for at least one day; and 3) any adverse reaction to the topical treatments applied in Arm 3 (lotion, ultrasound gel) such as rash or pruritis. Adverse events are reported twice annually to the Data and Safety Monitoring Board (DSMB). Furthermore, all SAEs are reported to the DSMB within 48 h of study staff becoming aware of the event. SAEs that are deemed ‘probably associated’ with the trial’s activities are also reported to the sIRB located at BWH within 48 h.

A protocol deviation is any noncompliance with or divergence from the sIRB-approved protocol that does not negatively affect the patient’s safety, welfare, or eligibility, or the integrity of the trial data [64]. A protocol violation is any noncompliance with or divergence from the sIRB-approved protocol that negatively affects patient safety, welfare, eligibility, or integrity of trial data [64]. Upon becoming aware of a deviation or violation, the site RC completes a protocol deviation/violation form in REDCap, assesses whether it is a deviation or violation, and sends the form to the site principal investigator and project manager for review. Once every year, all deviations are sent to the sIRB for review. Any protocol violations that occur are first sent to the overall study principal investigator and then to the DSMB within 48 h and the sIRB within one week.

## Discussion

The efficacy of PT for symptomatic degenerative meniscal tear has not been examined critically [15]. We have designed TeMPO to investigate the fundamental question: what aspects of a traditional physical therapy regimen (home strengthening/stretching, therapist interaction, and in-clinic therapist-directed strengthening and functional exercises/manual therapy/stretching) are effective in the setting of concomitant osteoarthritis and symptomatic meniscal tear? When completed, TeMPO will establish the efficacy of a therapist-directed strengthening and functional exercise-based program with manual therapy in clinic as comparison to: (1) placebo therapy overseen by a therapist and (2) unsupervised home-based strengthening exercise in addition to the other hypotheses outlined previously.

The trial design features many innovative components, including a placebo physical therapy intervention. To construct a robust placebo physical therapy intervention that is both feasible to perform and plausible to subjects, without conferring any significant physiologic benefit, we adapted approaches taken by Bennell and colleagues [18, 22, 65] as well as other placebo physical therapy interventions for osteoarthritis and other conditions. Many of these studies used placebo ultrasound and other physiologically inert components similar to our placebo regimen [16, 17, 19–21, 23–25]. We also included the application of placebo lotion aimed at mimicking the hands-on attention received from effleurage/therapeutic soft tissue mobilization, and placebo manual therapy, designed to mimic the hands-on aspects of true manual therapy without the associated physiologic benefits. In order to standardize regimen delivery, we created video demonstrations and hosted in-person meetings and numerous conference calls with all study therapists to ensure that each therapist was comfortable with the regimen and was appropriately trained.

The identification of symptomatic non-traumatic meniscal tear is a challenge for the TeMPO Trial. As 60 to 90% of persons with knee OA have concomitant meniscal tear on MRI, [2–6] identifying subjects whose tear contributes to knee pain is clinically complex. In the absence of a gold standard for determining whether a patient’s symptoms are due to meniscal tear, we called upon the clinical experience of enrolling physicians, who indicate whether the potential subject’s symptoms likely arise from meniscal tear (notably by the presence of mechanical symptoms including catching, locking, focal joint line tenderness, pain with internal/external rotation, and joint line pain with squatting) as was done in the MeTeOR trial [8].

A potential limitation of the TeMPO trial is the reliance on self-report data for adherence to the protocolized home exercise program. We encourage compliance by providing modest financial incentives to those completing the home exercise logs and questionnaires in order to maximize participation and minimize this limitation. We also assure subjects that their responses on the logs will not affect any aspect of their healthcare or trial participation. However, given different levels of trial engagement between the in-clinic arms and home exercise arms, the response rate may not be equivalent across arms. Also, the trial results will only be applicable to persons 45 years old and over with a degenerative meniscal tear electing to undergo non-operative management. Lastly, as with all research protocols we balanced the standardization of the intervention (e.g. a specific set of home exercises) with the clinical practice of tailoring intervention to subjects’ individual impairtments.

We have carefully considered several challenges in designing TeMPO, including therapist comfort with the placebo intervention, the identification of subjects with symptomatic meniscal tear, and patient adherence. While important clinical questions are rarely resolved by a single trial, the goal of TeMPO is to advance the understanding of the elements of a PT-based treatment that are most efficacious for symptomatic meniscal tear in the setting of mild to moderate knee osteoarthritis.

## Additional files

Additional file 1: Home exercise instructional pamphlet given to all subjects randomized in the TeMPO Trial. The pamphlet provides a detailed overview of the home exercise program with specific instructions regarding progression of exercise difficulty and knee pain while exercising. Each exercise to be completed is then broken down into step-by-step instructions with accompanying images. The home exercise program instructions are also available in a video format with the link provided in the home exercise instructional pamphlet. (PDF 1791 kb)

Additional file 2: Contains screenshots demoing the placebo manual therapy components of Arm III of the TeMPO trial. The potential procedures detailed are: Therapist Guided Hip Internal/External Rotation and Ankle Plantar/Dorsi Flexion and Inversion/Eversion. (DOCX 5028 kb)

Additional file 3: The exhaustive list of strengthening exercises with accompanying descriptions/instructions that could be performed under therapist supervision in the Arm IV in-clinic strengthening arm of the TeMPO trial. (DOCX 55 kb)

## Abbreviations

ACSM: American College of Sports Medicine
AE: Adverse Event
APM: Arthroscopic Partial Meniscectomy
APTA: American Physical Therapists Association
BWH: Brigham and Women’s Hospital
CC: Cleveland Clinic
DSMB: Data Safety Monitoring Board
KL: Kellgren-Lawrence
KOOS: Knee Osteoarthritis Outcomes Score
MRI: Magnetic Resonance Imaging
MSK: Musculoskeletal
OA: Osteoarthritis
OAPol: Osteoarthritis Policy Model
OARSI: Osteoarthritis Research Society International
PT: Physical Therapy
QALYs: Quality Adjusted Life Years
RC: Research Coordinator
RCT: Randomized Controlled Trial
REDCap: Research Electronic Data Capture
SAE: Serious Adverse Event
SD: Standard Deviation
sIRB: Single Institutional Review Board
SMS: Short Message Service
SPIRIT: Standard Protocol Items: Recommendations for Interventional Trials
TeMPO: Treatment of Meniscal Tear in Osteoarthritis (trial name)
TiDIER: Template for Intervention Description and Replication
TKR: Total Knee Replacement
UB-SMBS: University at Buffalo Jacobs School of Medicine and Biomedical Sciences
UPMC: University of Pittsburgh Medical Center
