# Supplementary material for: The TeMPO trial (treatment of meniscal tears in osteoarthritis): rationale and design features for a four arm randomized controlled clinical trial
Source: BMC Musculoskelet Disord. 2018 Dec 1;19:429. doi: 10.1186/s12891-018-2327-9 (PMC6271417; doi:10.1186/s12891-018-2327-9)
Supplement: Supplementary file 1 — Home exercise instructional pamphlet given to all subjects randomized in the TeMPO Trial. The pamphlet provides a detailed overview of the home exercise program with specific instructions regarding progression of exercise difficulty and knee pain while exercising. Each exercise to be completed is then broken down into step-by-step instructions with accompanying images. The home exercise program instructions are also available in a video format with the link provided in the home exercise instructional pamphlet. (PDF 1791 kb) [file 12891_2018_2327_MOESM1_ESM.pdf]

A Guide to your Home Exercises

Table of Contents

Welcome	
Item	Page Number
Introduction	ii
General instructions	iii
Exercise summary	iv
Using this pamphlet	v
Using your weights	vi
Exercise progression	vii
A note on pain	ix
**Exercises**	
Stretches	1-3
Strengthening	4-10
Functional	11-13
Exercise Logs	14-16

Introduction

Welcome to the **Tre**atment of **M**eniscal **P**roblems in **O**steoarthritis (**TeMPO**) study!

- This **home exercise guide** contains specific instructions to guide you in exercises we hope will strengthen your leg muscles and help relieve pain.
- The **Instructions** section (page iii) contains an overview of the complete exercise program. Please read it carefully.
- In addition to this packet, you should have also received a **USB Flash Drive** which includes the videos of physical therapy instructions, as well as an adjustable **10 lb. weight cuff set** in the mail. If you have not received either of these items, please contact us right away at the email or phone number below.
  - The instructional exercise videos can also be found at this link: http://oracore.bwh.harvard.edu/tempo-exercise-video/
    - Password: **exercises**
- Pages 14-16 of this pamphlet are **exercise logs**. Filling out the logs is optional, but we recommend using them to keep track of your exercise progress.
- *If you have any questions, do not hesitate to contact us at TeMPO@partners.org or 617-732-4313.*

General Instructions

Please watch all of the exercise videos we provided before attempting any of the exercises. While this pamphlet provides images and written directions for every exercise, it is important to watch the video first for a full understanding of how the exercises are performed.

Directions:

- **When you start the program, begin every exercise without any added weight.**
  - If you cannot do the exercise, switch to the *alternate* version of that exercise. Return to the primary exercise once you can do the alternate exercise easily and without any soreness or pain.
- **Complete the exercises on both legs.**
  - We recommend starting each exercise with the leg that is not painful so that you familiarize yourself with the activity first.
- **Focus on one exercise at a time.**
  - Complete all sets and repetitions of an exercise on both legs before moving on. For instance, finish your quadriceps exercises on both legs before beginning the hamstring exercises.
- **Go at your own pace.**
  - Take your time with the exercises, and do them at a comfortable speed to make sure you perform them correctly and complete the prescribed number of repetitions.
- **You can ice your knee for 5-10 minutes after each session if you feel sore.**
  - Do not apply the ice directly to your skin. Instead, place the cold pack in a pillowcase or dishtowel before placing it on your skin.

Always remember!

*If you experience severe **pain** or **cramping** at any point, **stop** immediately and rest.*

Exercise Summary

Below is a comprehensive list of the exercises included in this packet:

Exercise Type	Primary Exercise Name	Target Muscle	Notes
Stretch	Hamstring Stretch	Hamstrings	No alternate exercise
	Side Lying Quadriceps Stretch	Quadriceps	Alternate exercise: Standing Quadriceps Stretch
Strengthening	Bent Over Hip Extension*	Gluteus Maximus	Alternate exercise: Bridging
	Side Lying Leg Lift*	Gluteus Medius	Alternate exercise: Clamshell
	Straight Leg Raise*	Quadriceps	Alternate exercise: Seated Knee Extension
	Standing Hamstring Curl*	Hamstrings	No alternate exercise
Functional	Squats	Multiple	3 levels (progress at your own pace): Initial: Mini Wall Squats Intermediate: Regular Chair Squats Advanced: Staggered Chair Squats

*Exercise can be made more challenging by adding weight (see page vi).

Where are the muscles located?

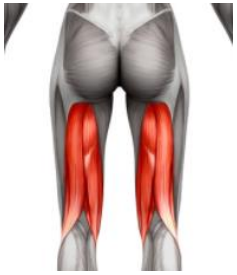

Hamstrings
Behind the thigh

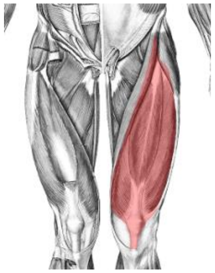

Quadriceps
In front of the thigh

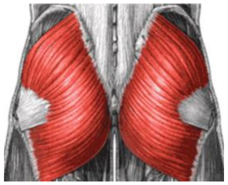

Gluteus Maximus
Behind the hip

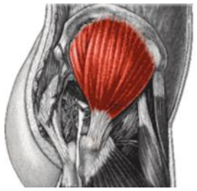

Gluteus Medius
Outside of the hip

Perform each <u>stretch</u> twice, holding for 30 seconds each time
Perform each <u>strengthening and functional exercise</u> for 3 sets of 8-12 repetitions

Using this pamphlet

At the top of each exercise page, you will notice a header that looks similar to this:

**Exercise type:** Stretch	**Target muscle:** Quadriceps	Primary exercise

Each of these categories contains important information about your exercises. Find the meanings below:

Exercise Type:

Stretching: Stretching exercises will help increase your flexibility and range of motion, which is important for recovery and future injury prevention.

Strengthening: These exercises are designed to strengthen specific muscles in a targeted manner.

Functional: Functional exercises are also strengthening, but do not target individual muscles. Instead, they strengthen multiple muscle groups at once and increase your range of motion to help you perform typical daily activities with greater ease and comfort.

Muscle Group:

Hamstrings: A large muscle at the back of your thigh

Quadriceps: A large muscle at the front of your thigh

Gluteus Maximus: The largest muscle in your buttocks

Gluteus Medius: Another muscle in your buttocks, located near the large hip bone on your side

Primary or Alternate Exercise:

Primary Exercise: Begin with the primary exercises without any weight and assess the difficulty level. If you feel comfortable, focus on these exercises as you progress through the home exercise program.

Alternate Exercise: If the primary exercises are too difficult or are causing you pain, opt for the alternate exercises. These still target the same muscle, but may be more comfortable for you. Once you can easily perform all of the repetitions of an alternate exercise, return to the primary exercise.

Using your weights

If you can easily perform 3 sets of 12 repetitions for an exercise and you don't have any soreness two days after your exercise, you may increase the difficulty of some exercises by adding additional weight.

To use your weights:

1. Insert the desired amount of weight into your weight cuff. Each small bar weighs **0.5 pounds.**
2. Wrap the weight cuff securely around your ankle. When secured, it should stay in one place while you move, without sliding up or down.
3. Make sure the weight cuff does not restrict your motion.
4. Perform the exercise the same way as you would without the weights. Be extra careful of your exercise form!
5. We recommend adding one pound at a time.

You can add weight to the following 4 exercises:
Bent Over Hip Extension | Side Lying Leg Lift | Straight Leg Raise | Standing Hamstring Curl

Example: Using weights with the Bent Over Hip Extension

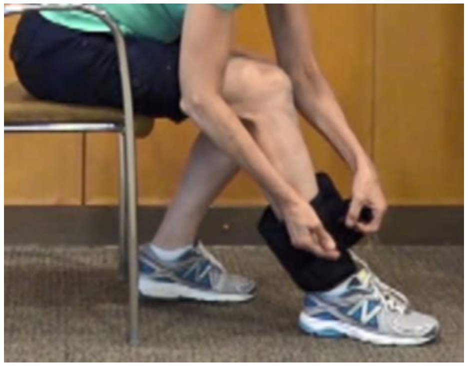

Adding weight

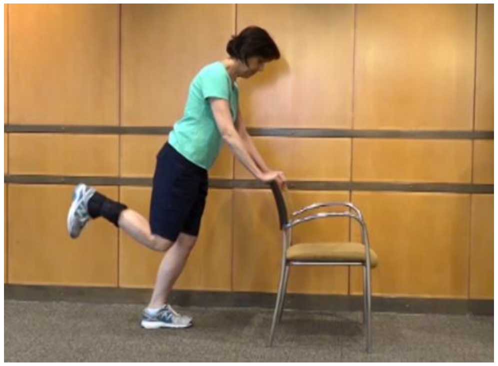

Starting position

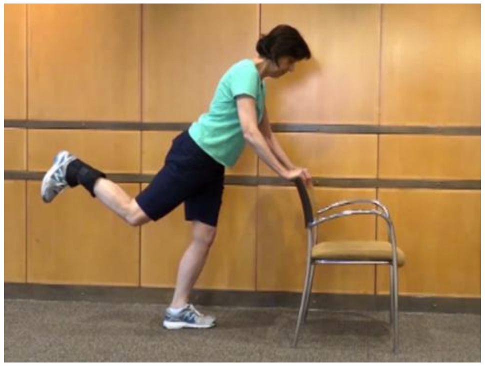

Ending position

Exercise Progression

Each exercise can be tailored to your own ability and strength. Generally, exercise intensity level can be increased in two ways: **increasing repetitions** or **adding weight.**

To move through the home exercise program, follow these guidelines:

1	2
Start with **3 sets of 8 repetitions** of the exercises with **no weight** for each muscle group.	Increase your repetitions up to **3 sets of 12 repetitions** if an exercise feels easy.<br>• An exercise is "easy" if you feel as if you could do an additional 4-6 more repetitions after completing the final set.<br>• It is okay if an exercise never feels easy – simply stay at 8 repetitions.

Exercise Progression, continued

3

If 3 sets of 12 repetitions becomes very easy for an exercise, you may begin performing that exercise with **added weight**.

a. Start by adding 1-2 lbs., and return to 3 sets of 8 repetitions. Increase to 12 repetitions per set when you feel comfortable.
b. Progress by adding weights in **1 lb. increments**. Remember: only add weight if the exercise becomes easy, and you do not experience any soreness.
c. Different exercises can progress at different speeds.
   - For instance, you can be doing 3 sets of 10 repetitions of the gluteus maximus exercise with no added weight, but 3 sets of 8 repetitions of quadriceps exercise with an additional 2 lbs.

Remember:

1. Do not increase repetitions or difficulty if you experience **knee pain** for more than **24 hours** after exercise or are **too fatigued** to complete a set.

2. If an exercise is too difficult or you feel intense pain, switch to the **alternate** exercise and complete 3 sets of 8-12 repetitions, following the same guidelines as above for progression.

A note on pain

The TeMPO exercises will help increase the strength of the muscles around your knee. However, strengthening muscles can be a difficult and sometimes painful process. Differentiating between "okay" soreness in your leg muscles and "not okay" pain that can be associated with injury can be difficult.

We have assembled a few tips to help you understand how far to push yourself in these exercises.

Okay

•Dull or aching pain and fatigue during the final repetitions of an exercise

•Mild soreness up to 48 hours after exercising when doing daily activities, such as walking

•Mild throbbing or warmth in the muscles worked up to 24 hours after exercise

•Mild tenderness in the **muscles** worked up to 48 hours after exercising

•Mild swelling of the muscles worked

Not Okay

•Acute, sharp, or stabbing pain during the exercise

•Severe and sudden muscle cramping inhibiting movement during or after exercise

•Any ripping, popping, or tearing sounds

•Buckling of the knee or collapsing

•Uncomfortable throbbing sensations more than 24 hours after exercising

•Tenderness and weakness in the **knee** more than 48 hours after exercising

•Severe soreness inhibiting basic daily activities

If you experience any of the items listed in the "Not Okay" column, stop the exercise. If the pain continues, contact your physician

The

TeMPO

Exercises

Exercise type: Stretch | **Target muscle:** Hamstring | Primary exercise

Hamstring Stretch

To start:

Find a stable surface, such as your bed or the floor, and lie comfortably on your back.

The stretch:

1. Firmly grasp your thigh with both hands and bend your hip and knee at a 90-degree angle.

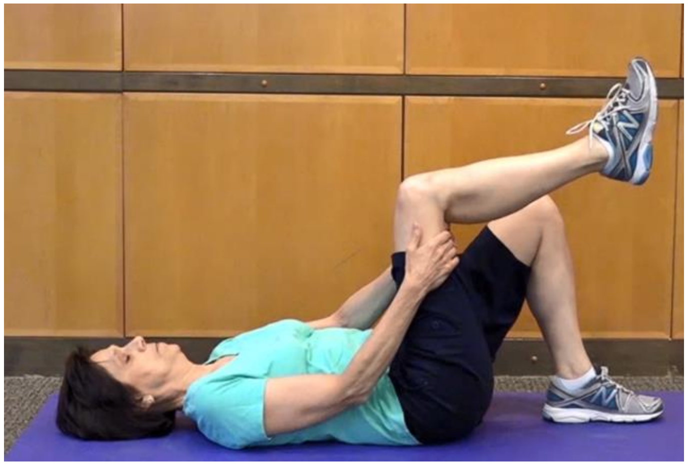

2. Slowly straighten your leg as much as possible.

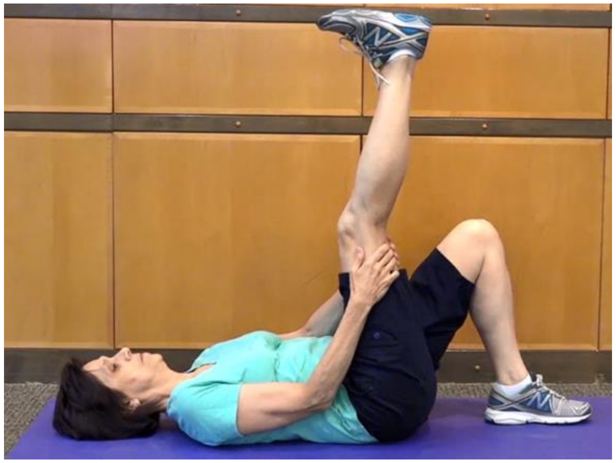

Tip: If you cannot comfortably grab your thigh, use a towel to grasp your thigh.

Hold for 30 seconds
Repeat 2-3 times for each leg

If there is severe pain or cramping, stop the stretch immediately

Exercise type: Stretch | **Target muscle:** Quadriceps | Primary exercise

Side Lying Quadriceps Stretch

To start:

Find a stable surface, such as your bed or the floor, and lie comfortably on your side.

The stretch:

1. Bend your top leg.

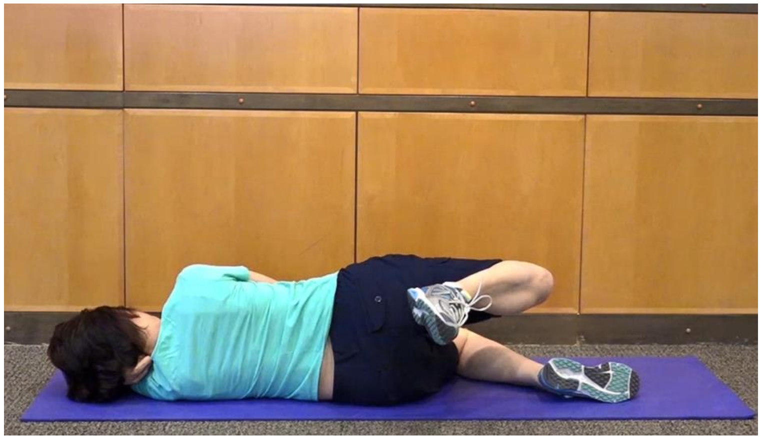

2. Grab your top ankle with your top arm.

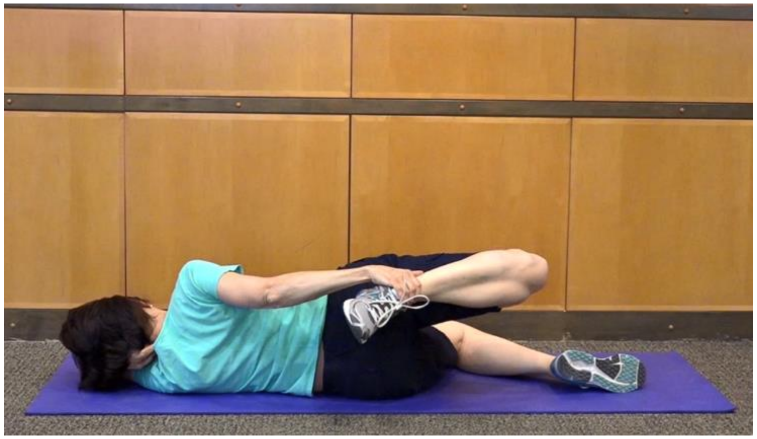

Tip: Your bottom leg can be either bent or straight. Make sure to keep your top leg parallel to your body, and keep your body in line facing forward.

Hold for 30 seconds
Repeat 2-3 times for each leg

If there is severe pain or cramping, stop the stretch immediately

Exercise type: Stretch | **Target muscle:** Quadriceps | Alternate exercise

Standing Quadriceps Stretch

To start:

Perform this exercise if you cannot do the Side Lying Quadriceps Stretch.
Hold onto a stable surface, such as a chair, table or wall.

The stretch:

1. Stand in a relaxed position with your feet shoulder-width apart.

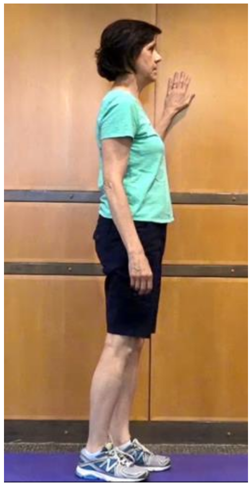

2. Grab your ankle and pull straight back towards your buttocks.

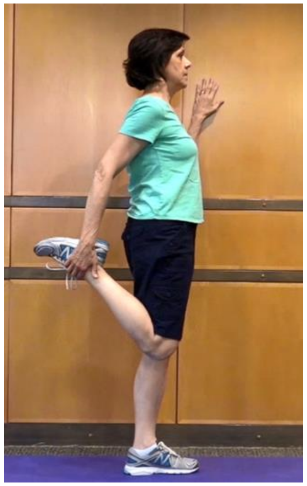

Tip: Make sure to stand up tall. Try not to bend your back or lean forward. Take care to be safely balanced.

Hold for 30 seconds
Repeat 2-3 times for each leg

If there is severe pain or cramping, stop the stretch immediately

Exercise type: Strengthening | **Target muscle:** Gluteus Maximus | Primary exercise

Bent Over Hip Extension

To start:

Rest your hands on a stable surface, such as a table, chair or kitchen countertop. Lean against it, slightly bending at the waist, with feet shoulder width apart.

The exercise:

1. Bend your exercising leg.

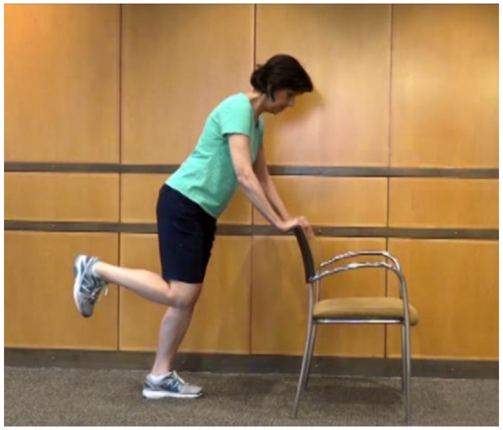

2. Gently lift your leg up from the hip, keeping the knee bent, and push up towards the ceiling in a controlled manner. Then lower the leg back down.

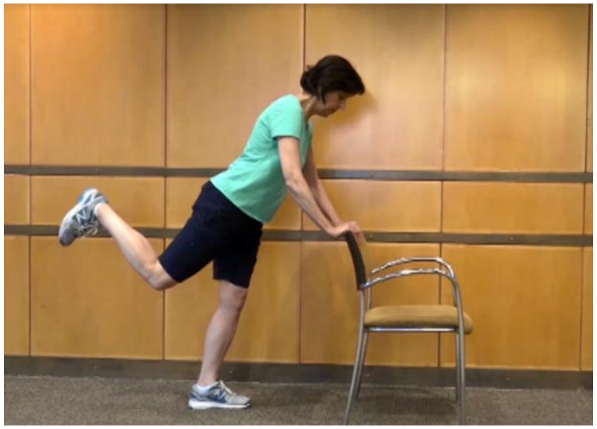

Tip: Make sure your exercising leg stays in line with your body, without rotating inwards or outwards.

Complete 3 sets of 8-12 repetitions for each leg

If you can complete all the sets and repetitions easily with no pain or soreness, use the **weights** provided and slowly add weight (1 lb. at a time) for an added challenge.

If there is severe pain or cramping, stop the exercise immediately

Exercise type: Strengthening | Target muscle: Gluteus Maximus | Alternate exercise

Bridging

To start:

Perform this exercise if you cannot do the Bent Over Hip Extension.
Lay down on a yoga mat, carpet, or a firm bed.

The exercise:

1. Bend your knees.

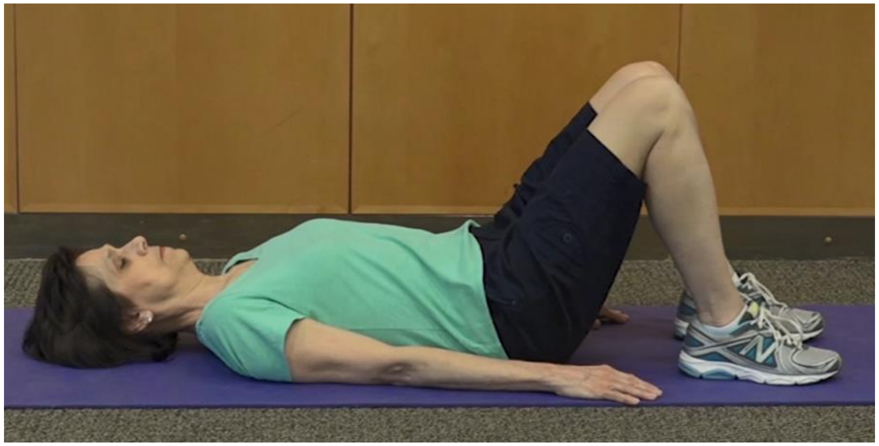

2. Lift your buttocks, raising your hips as high as you can towards the ceiling. Hold for 3-5 seconds before lowering down to starting position.

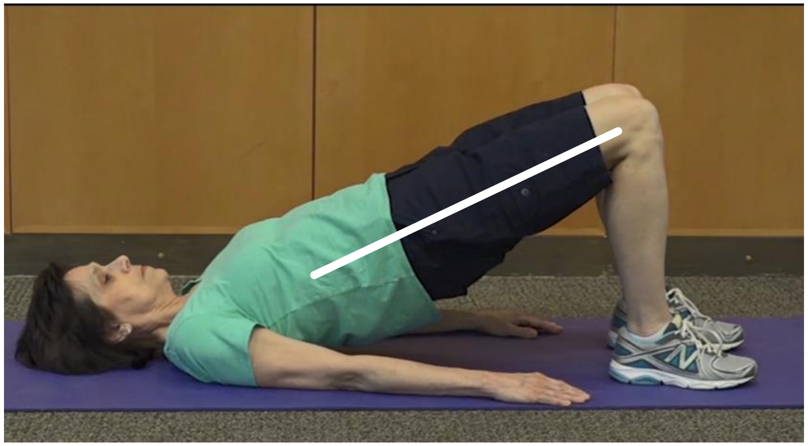

Tip: Make sure you do not arch your back. Arching may be prevented by squeezing your buttocks and tucking your tailbone.

Complete 3 sets of 8-12 repetitions

If there is severe pain or cramping, stop the exercise immediately

Exercise type: Strengthening | **Target muscle:** Gluteus Medius | Primary exercise

TeMPO

Side Lying Leg Lift

To start:

Find a stable surface, such as your bed or the floor, and lie comfortably on your side.

The exercise:

1. Find a stable starting position on your side with your top leg in line with your shoulders and your top knee straight.

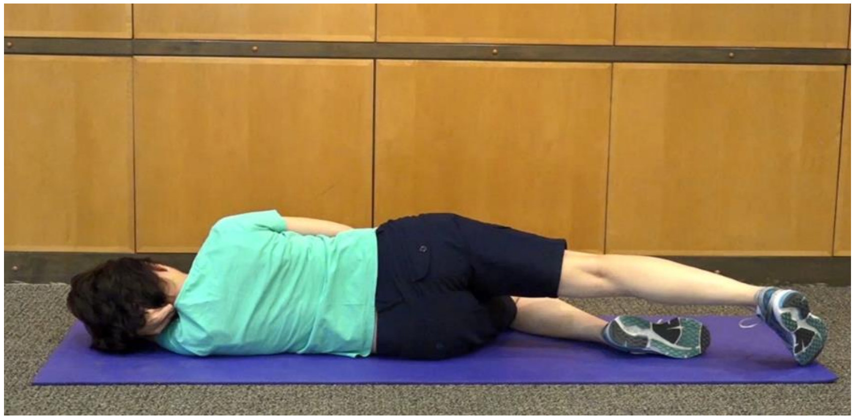

2. Slowly lift your top leg, leading with your heel. Hold for 3-5 seconds at the top, then lower slowly.

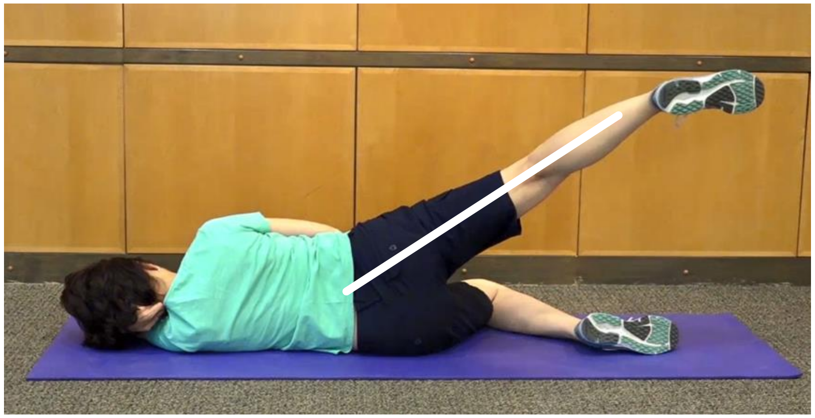

Tip: Keep your body in a straight line throughout the exercise. Your bottom leg can be bent or straight. Do not allow the top leg to swing forward or to rotate.

Complete 3 sets of 8-12 repetitions for each leg

If you can complete all the sets and repetitions easily with no pain or soreness, use the **weights** provided and slowly add weight (1 lb. at a time) for an added challenge.

If there is severe pain or cramping, stop the exercise immediately

Exercise type: Strengthening | **Target muscle:** Gluteus Medius | Alternate exercise

Clamshell

To start:

Perform this exercise if you cannot do the Side Lying Leg Lift.
Find a stable surface, such as your bed or the floor, and lie comfortably on your side.

The exercise:

1. Keep your hip straight (in line with your shoulders), and bend your knees at a 90-degree angle.

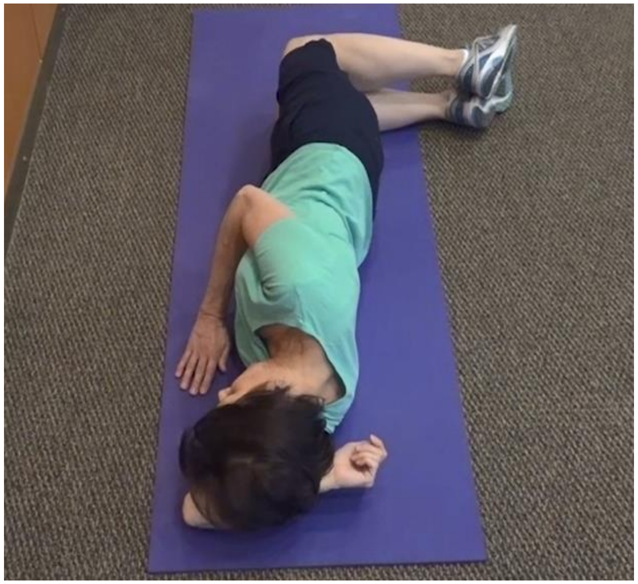

2. Keeping your heels together, raise your top knee away from the bottom knee without moving the rest of your body. Hold for 3-5 seconds at the top, then lower slowly.

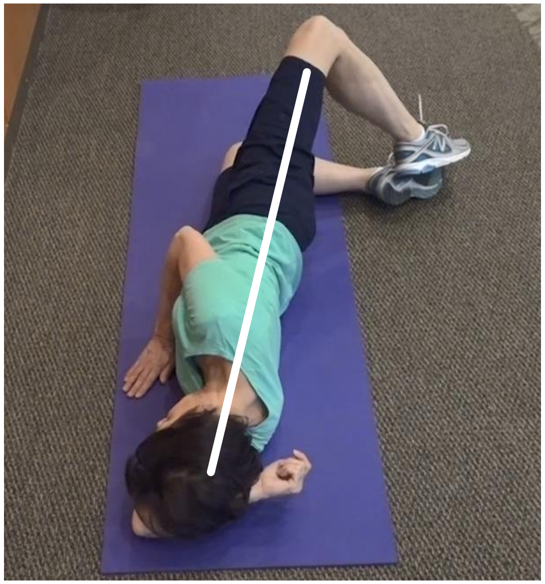

Tip: Keep your body in a straight line. You should feel the muscle working in your buttocks.

Complete 3 sets of 8-12 repetitions

If there is severe pain or cramping, stop the exercise immediately

Exercise type: Strengthening | **Target muscle:** Quadriceps | Primary exercise

Straight Leg Raise

To start:

Find a stable surface, such as your bed or the floor, and lie comfortably on your back.

The exercise:

1. Bend your non-exercising knee, and rest your exercising leg straight on the floor.

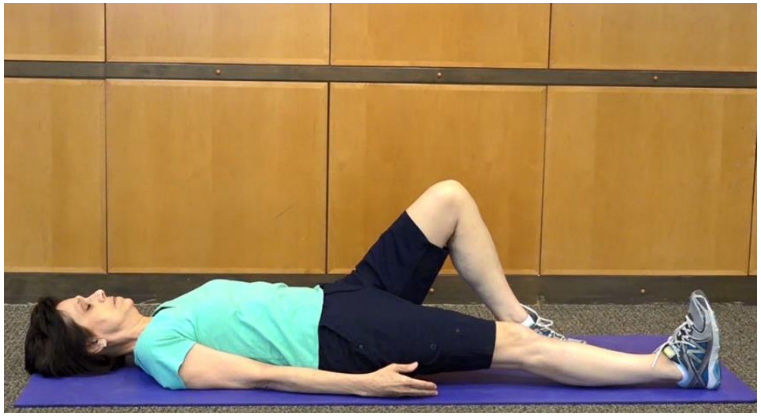

2. Tighten your thigh muscle and pull your toes back. Keeping your leg straight, slowly raise your leg to the same angle as your bent leg. Hold 3-5 seconds, then slowly lower the leg down to the floor.

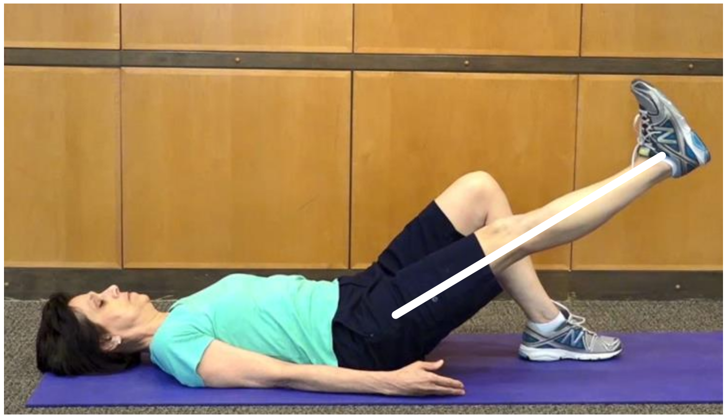

Tip: Make sure to keep your exercising knee straight, and try not to let your leg drift out to the sides.

Complete 3 sets of 8-12 repetitions for each leg

If you can complete all the sets and repetitions easily with no pain or soreness, use the **weights** provided and slowly add weight (1 lb. at a time) for an added challenge.

If there is severe pain or cramping, stop the exercise immediately

Exercise type: Strengthening | **Target muscle:** Quadriceps | Alternate exercise

Seated Knee Extension

To start:

Perform this exercise if you cannot do the Straight Leg Raise.
Sit with your back and thighs comfortably supported in a stable chair.

The exercise:

1. Rest your feet flat on the floor at a 90-degree angle.

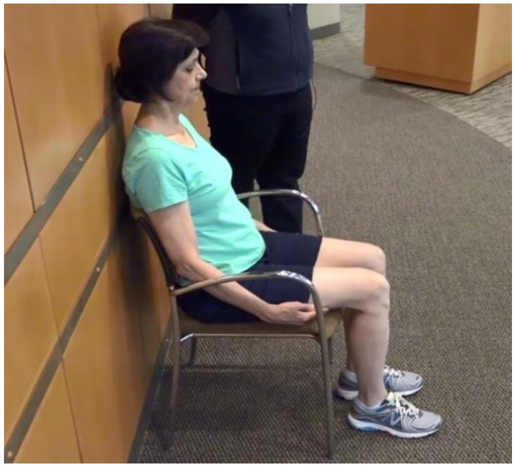

2. Lift your heel to straighten the knee, extending your leg to be parallel with the floor. Hold for 3-5 seconds, then lower slowly.

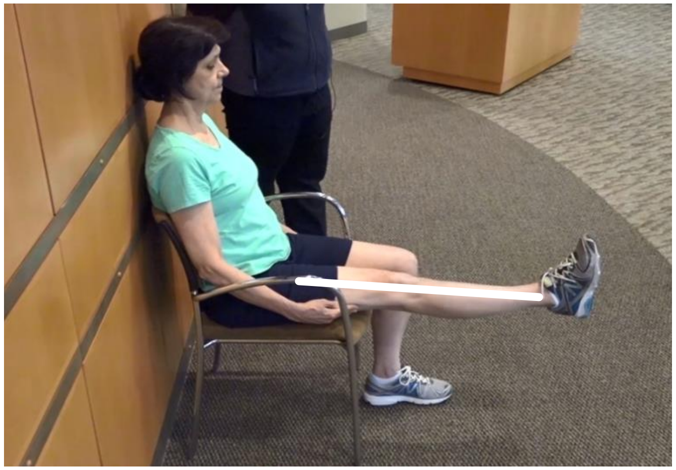

If you can complete all the sets and repetitions easily with no pain or soreness, use the **weights** provided. Slowly add weight (1 lb. at a time) for an added challenge.

Complete 3 sets of 8-12 repetitions for each leg

If there is severe pain or cramping, stop the exercise immediately

Exercise type: Strengthening | **Target muscle:** Hamstring | Primary exercise

Standing Hamstring Curl

To start:

Stand with your hands supported on a wall, countertop, or other stable surface.

The exercise:

1. Stand up straight, with your body in a relaxed, neutral position.

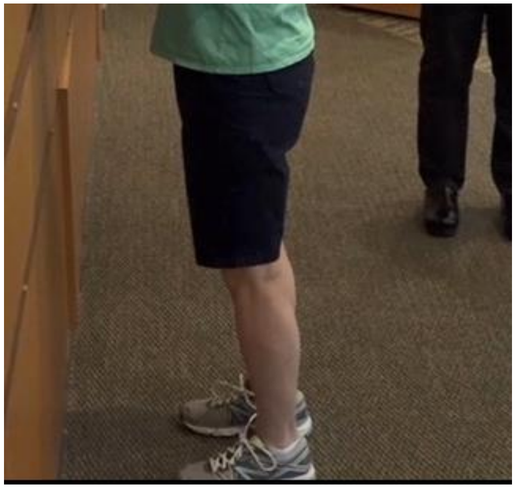

2. Bend your knee back towards your buttocks, keeping your thigh in line with the non-exercising leg.

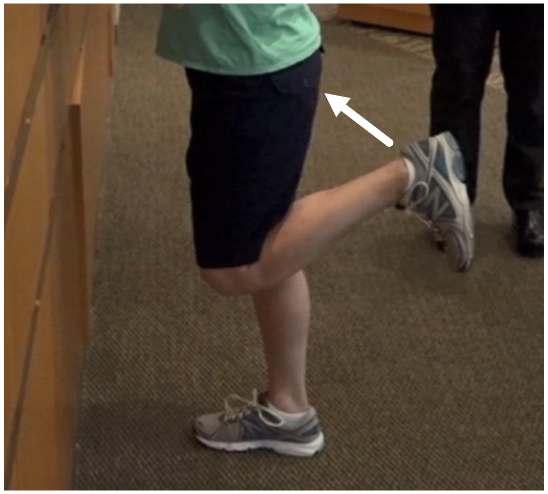

Tip: Keep your leg behind you, and make sure not to let the leg rotate to the left or right. Stand up straight and avoid bending your body forward.

Complete 3 sets of 8-12 repetitions for each leg

If you can complete all the sets and repetitions easily with no pain or soreness, use the **weights** provided and slowly add weight (1 lb. at a time) for an added challenge.

If there is severe pain or cramping, stop the exercise immediately

Exercise type: Functional | **Target Muscle:** Multiple | **Level**: Initial

TeMPO

Mini Wall Squat

To start:

Stand with your feet shoulder-width apart.

The exercise:

1. Stand up straight, with your body in a relaxed, neutral position. The distance between you and the wall should be about the length of one shoe.

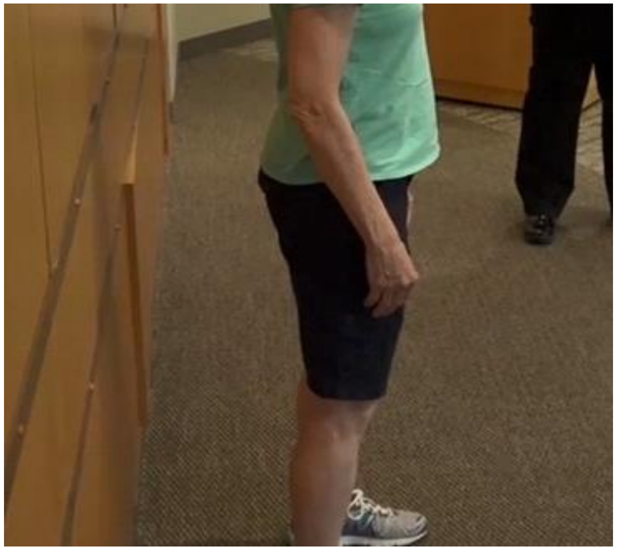

2. Gently bend your knees, lowering your buttocks towards the ground while keeping your knees in line with your ankles. Hold for 3-5 seconds before rising back up slowly.

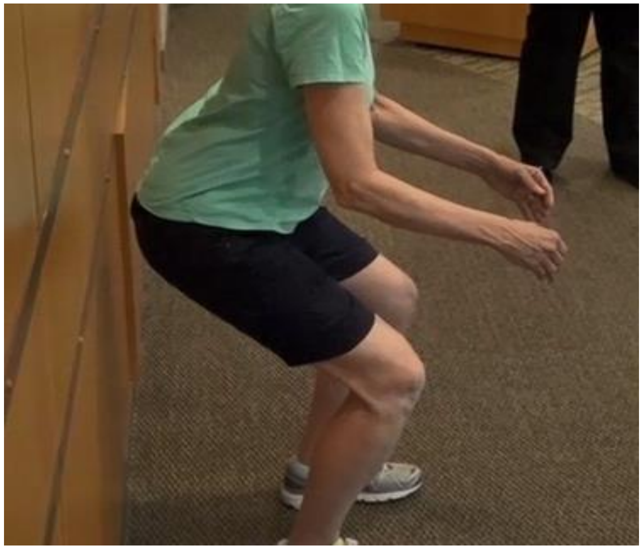

Tip: Make sure you keep your heels on the ground. Also, do not let your knees go forward past your toes, do not let your knees collapse inward, and do not bend your knees more than 90 degrees.

Complete 3 sets of 8-12 repetitions

If there is severe pain or cramping, stop the exercise immediately

Exercise type: Functional | **Target Muscle:** Multiple | **Level:** Intermediate

Regular Chair Squat

To start:

Perform this exercise if you can complete 3 sets of 12 Mini Wall Squats easily.
Place a stable chair behind you. If possible, perform this exercise on a carpet with the chair against a wall.

The exercise:

1. Stand a few inches in front of the chair with your feet parallel.

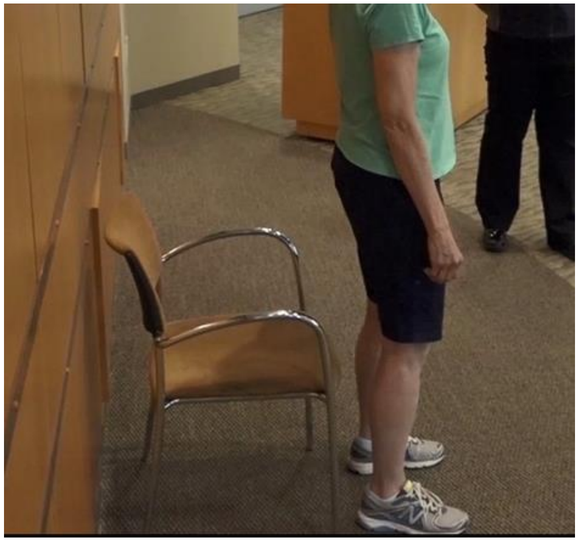

2. Gently bend your knees, lowering your hips so that your buttocks almost touch the chair, but do not sit down. Hold for 3-5 seconds before rising back up slowly.

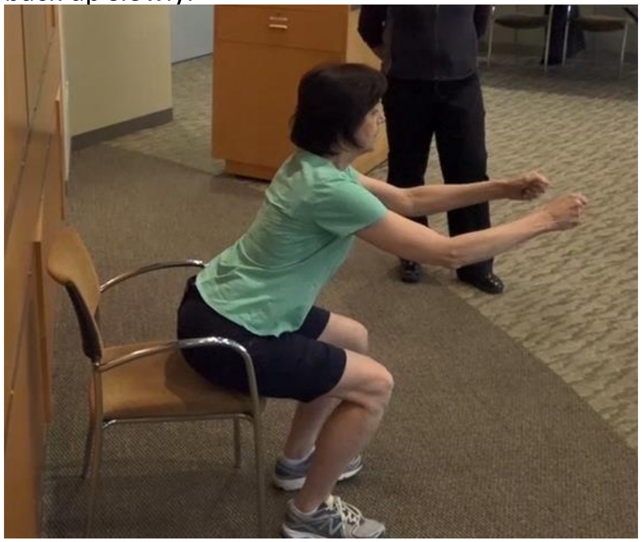

Tip: Keep your back as straight as possible with your belly pulled in tight and your knees parallel. Do not let your knees go forward past your toes. When you rise back up, you can use your hands for balance, but try not to use them to push yourself back up.

Complete 3 sets of 8-12 repetitions

If there is severe pain or cramping, stop the exercise immediately

Exercise type: Functional | **Target Muscle:** Multiple | **Level:** Advanced

Staggered Chair Squat

To start:

Perform this exercise if you can complete 3 sets of 12 Regular Chair Squats easily.
Place a stable chair behind you. If possible, perform this exercise on a carpet with the chair against a wall.

The exercise:

1. Stand up straight and stagger your legs so that your exercising leg is about 6 inches behind your non-exercising leg.

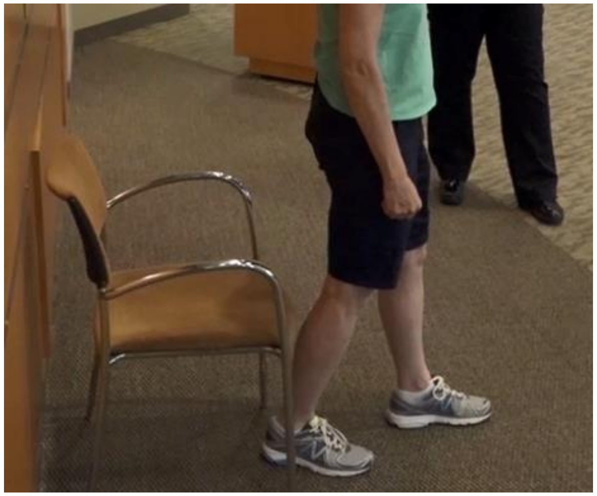

2. With your exercising leg pointed out, gently sit back so that your buttocks almost touch the chair, but do not sit down. Hold for 3-5 seconds before rising back up slowly.

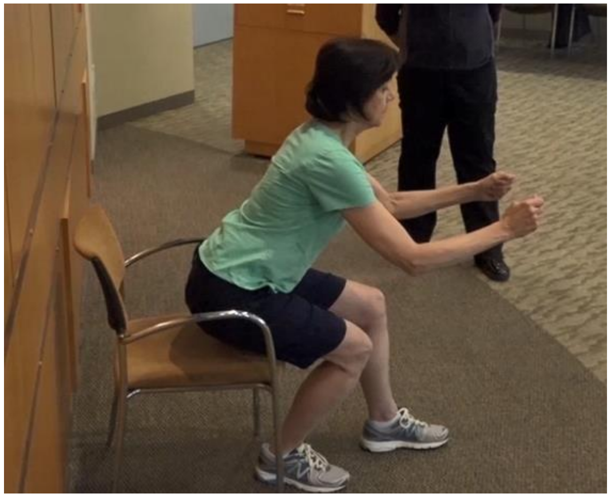

Tip: Most of your weight should be on the leg that is behind you. Keep your legs strong, and do not let your exercising knee collapse inward or go beyond your toes.

Complete 3 sets of 8-12 repetitions

If there is severe pain or cramping, stop the exercise immediately

Please use the table below to keep track of your exercises and progress. We recommend checking ✓ off the days you exercised and writing notes (such as exercise performed, reps, sets and weights) that will help you stick to your exercise routine. This may also be useful to reference when you fill out the study logs we will send to you every other week.

Exercise Log: Month 1

	Monday	Tuesday	Wednesday	Thursday	Friday	Saturday	Sunday
Week 1	Date: ________ ☐	Date: ________ ☐	Date: ________ ☐	Date: ________ ☐	Date: ________ ☐	Date: ________ ☐	Date: ________ ☐
Week 2	Date: ________ ☐	Date: ________ ☐	Date: ________ ☐	Date: ________ ☐	Date: ________ ☐	Date: ________ ☐	Date: ________ ☐
Week 3	Date: ________ ☐	Date: ________ ☐	Date: ________ ☐	Date: ________ ☐	Date: ________ ☐	Date: ________ ☐	Date: ________ ☐
Week 4	Date: ________ ☐	Date: ________ ☐	Date: ________ ☐	Date: ________ ☐	Date: ________ ☐	Date: ________ ☐	Date: ________ ☐

Please use the table below to keep track of your exercises and progress. We recommend checking ✓ off the days you exercised and writing notes (such as exercise performed, reps, sets and weights) that will help you stick to your exercise routine. This may also be useful to reference when you fill out the study logs we will send to you every other week.

Exercise Log: Month 2

	Monday	Tuesday	Wednesday	Thursday	Friday	Saturday	Sunday
Week 5	Date: ______ ☐	Date: ______ ☐	Date: ______ ☐	Date: ______ ☐	Date: ______ ☐	Date: ______ ☐	Date: ______ ☐
Week 6	Date: ______ ☐	Date: ______ ☐	Date: ______ ☐	Date: ______ ☐	Date: ______ ☐	Date: ______ ☐	Date: ______ ☐
Week 7	Date: ______ ☐	Date: ______ ☐	Date: ______ ☐	Date: ______ ☐	Date: ______ ☐	Date: ______ ☐	Date: ______ ☐
Week 8	Date: ______ ☐	Date: ______ ☐	Date: ______ ☐	Date: ______ ☐	Date: ______ ☐	Date: ______ ☐	Date: ______ ☐

Please use the table below to keep track of your exercises and progress. We recommend checking ✓ off the days you exercised and writing notes (such as exercise performed, reps, sets and weights) that will help you stick to your exercise routine. This may also be useful to reference when you fill out the study logs we will send to you every other week.

Exercise Log: Month 3

	Monday	Tuesday	Wednesday	Thursday	Friday	Saturday	Sunday
Week 9	Date: ________ ☐	Date: ________ ☐	Date: ________ ☐	Date: ________ ☐	Date: ________ ☐	Date: ________ ☐	Date: ________ ☐
Week 10	Date: ________ ☐	Date: ________ ☐	Date: ________ ☐	Date: ________ ☐	Date: ________ ☐	Date: ________ ☐	Date: ________ ☐
Week 11	Date: ________ ☐	Date: ________ ☐	Date: ________ ☐	Date: ________ ☐	Date: ________ ☐	Date: ________ ☐	Date: ________ ☐
Week 12	Date: ________ ☐	Date: ________ ☐	Date: ________ ☐	Date: ________ ☐	Date: ________ ☐	Date: ________ ☐	Date: ________ ☐

Thank you for participating in

TeMPO

We hope you keep exercising!
