# Supplementary material for: The TeMPO trial (treatment of meniscal tears in osteoarthritis): rationale and design features for a four arm randomized controlled clinical trial
Source: BMC Musculoskelet Disord. 2018 Dec 1;19:429. doi: 10.1186/s12891-018-2327-9 (PMC6271417; doi:10.1186/s12891-018-2327-9)

Below are screenshots of the different procedures used in the Sham Physical Therapy Arm of the TeMPO Trial.

**Therapist Guided Hip Internal/External Rotation**


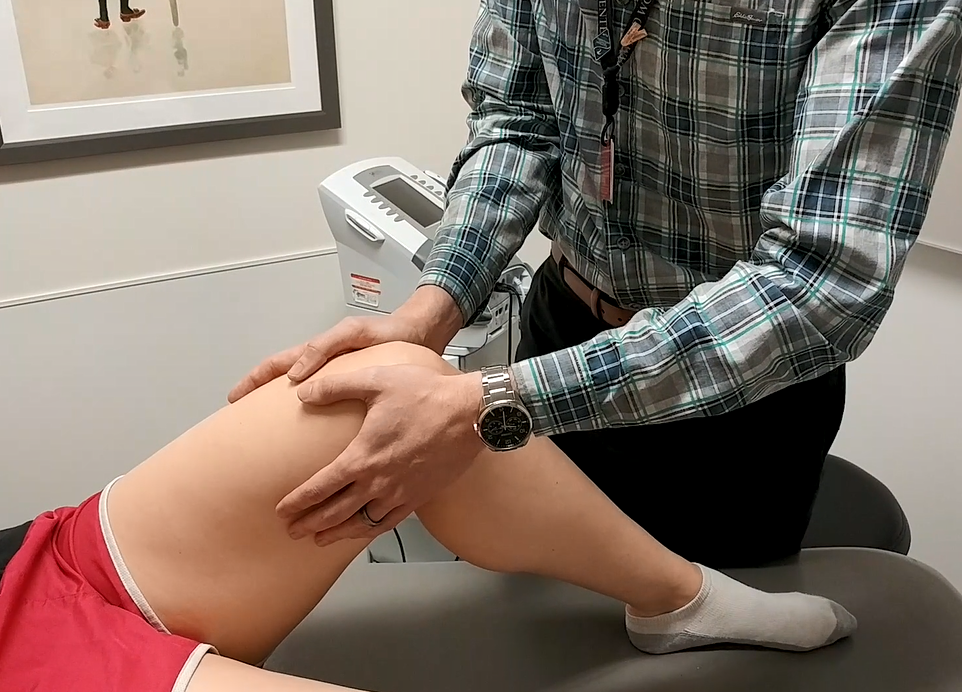


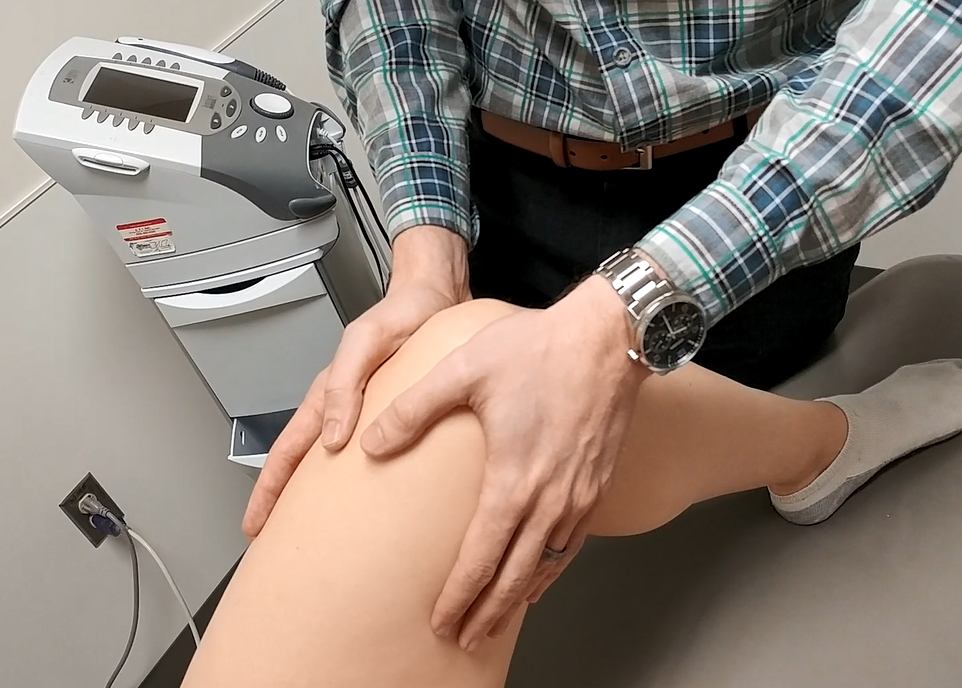


**Ankle Plantar/Dorsi Flexion and Inversion/Eversion**


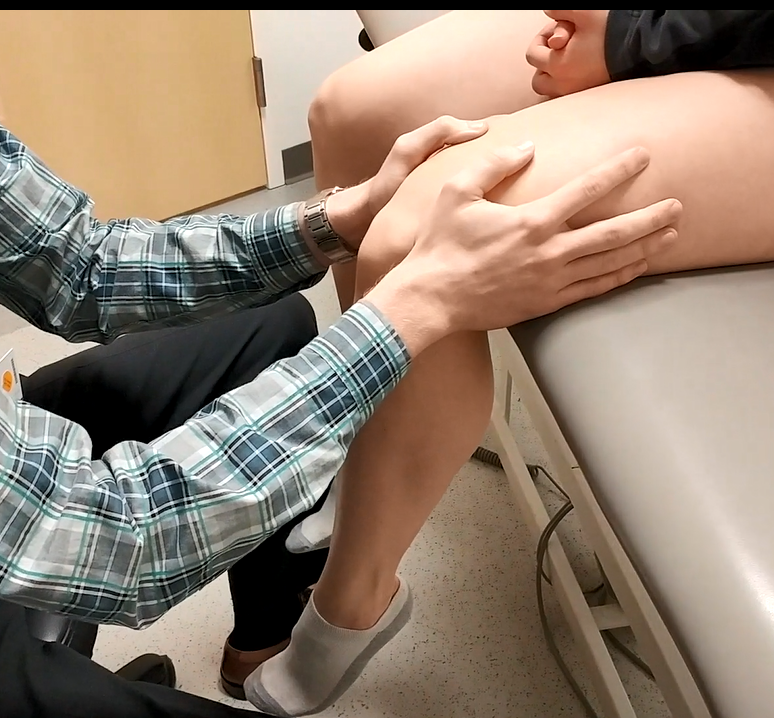


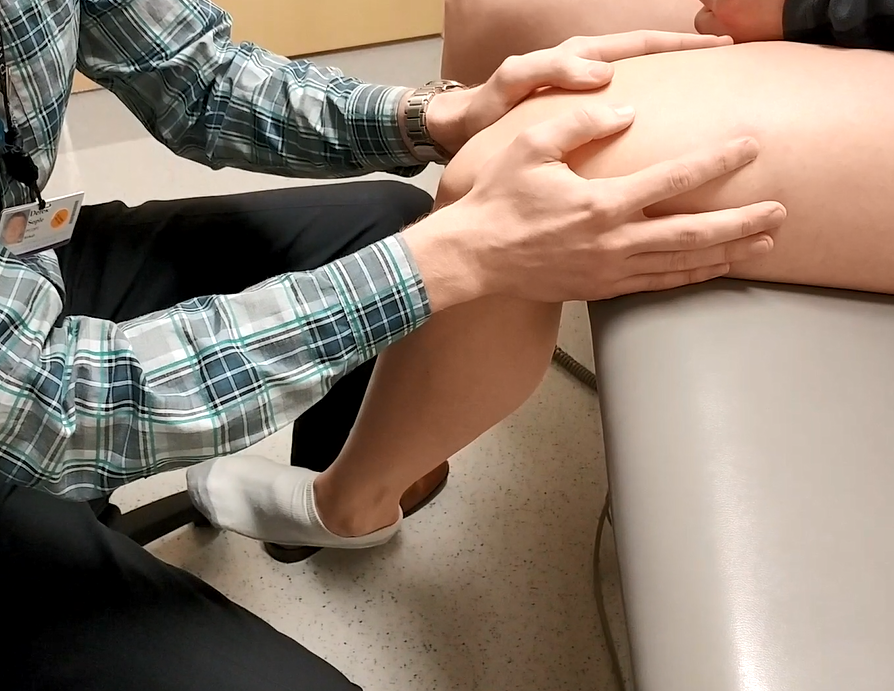

Supplement: Supplementary file 2 — Contains screenshots demoing the placebo manual therapy components of Arm III of the TeMPO trial. The potential procedures detailed are: Therapist Guided Hip Internal/External Rotation and Ankle Plantar/Dorsi Flexion and Inversion/Eversion. (DOCX 5028 kb) [file 12891_2018_2327_MOESM2_ESM.docx]
