# Supplementary material for: The TeMPO trial (treatment of meniscal tears in osteoarthritis): rationale and design features for a four arm randomized controlled clinical trial
Source: BMC Musculoskelet Disord. 2018 Dec 1;19:429. doi: 10.1186/s12891-018-2327-9 (PMC6271417; doi:10.1186/s12891-018-2327-9)
Supplement: Supplementary file 3 — The exhaustive list of strengthening exercises with accompanying descriptions/instructions that could be performed under therapist supervision in the Arm IV in-clinic strengthening arm of the TeMPO trial. (DOCX 55 kb) [file 12891_2018_2327_MOESM3_ESM.docx]

**Physical Therapy Exercises MOOP**

**Gluteus Maximus**

*Bent Over Hip Extension*

Find a stationary tabletop or the back of a chair or couch. Brace yourself with both hands and bend over slightly. Flex one knee, and slowly push the foot of the flexed knee towards the ceiling. Keep hips facing forward, do not bend left or right.

*Gluteus Maximus Kickback*

Place your hands on a supportive surface in front of you. Stand on one leg with the other knee bent, then extend your knee by straightening your leg. Slowly return to the starting position.

*Standing Hip Extension*

Hold on to a stable surface such as a chair, table or couch. With an elastic band attached at your ankle, draw your leg back. Keep your knee straight the entire time.

*Double Leg Bridge on Stable Surface*

Find a stable surface such as a couch, table or floor. Lie comfortably on your back with your knees bent. Slowly push your hips towards the ceiling. Stop when your knees, hips and shoulder form a straight line. Hold briefly, then slowly lower your hips back down to the starting position.

*Double Leg Bridge on Yoga Ball*

Start in a seated position on the ball. Next, slowly walk your feet forward so that the ball is on your upper back. Keep your buttocks and pelvis up off the ball and straight with your thighs. Hold briefly, then slowly return to the seated position.

*Straight Leg Bridge on Yoga Ball*

Find a stable surface such as a couch, table or floor. Lie comfortably on your back and place your feet on the yoga ball. Slowly push your hips towards the ceiling. Stop when your knees, hips and shoulders form a straight line. Hold briefly, then slowly lower your hips back down to the starting position.

*Single Leg Bridge*

While lying on your back, raise your hips off the floor into a bridge position. Next straighten a leg so that only one leg is supporting your body. Then, return that leg back to the ground and change to the other side. Try and keep your pelvis level the entire time.

**Gluteus Medius**

*Side-lying Straight Leg Lift*

Lie comfortably on your side on a stable surface with your bottom leg slightly bent and the top leg straight. Keeping the body still and the knee straight, slowly lift the top leg. Raise with the heel first, do not allow the top leg to swing forwards or rotate. Hold briefly, then lower slowly.

*Clam*

Lie on your side with your hips and knees slightly bent. Your knees and ankles should be touching, and your shoulders and hips should be in a straight line. Keeping the body still and your ankles together, slowly lift your top knee away from the bottom knee. Hold briefly then lower slowly.

*Standing Hip Abduction*

Stand with a supportive surface next to your side, hold on to the supportive surface with one hand. While standing with an elastic band looped around your ankles, move your leg out to the side as shown. Keep your hip, knee and ankle in a straight line.

*Gluteus Medius Kick Back*

Lie on a supportive surface on your side. For the leg that is on top, bend your knee and hip to move your leg forward. Then extend your hip and knee backward and diagonally upwards. Return leg to forward position.

*Standing Hip Hike*

While standing up on a step, lower one leg downward towards the floor by tilting your pelvis to the side. Then return the pelvis/leg back to a leveled position. Keep hips and shoulders in a straight line throughout.

*Isometric Wall Press*

Stand with both feet directly under your hips next to a wall. You can bring your arm in front of you so it does not get in the way. Next, bend the knee closest to the wall and push into the wall as if you were trying to push your body away, but without moving. Hold briefly, then return your foot to the ground.

*Lateral Band Walk*

With an elastic band around both ankles, walk to the side while keeping your feet spread apart. Keep your knees bent the entire time.

*Bridging with Band Around Knees*

While lying on your back, place an elastic band around your knees and pull your knees apart. Slowly push your hips towards the ceiling. Stop when your knees, hips and shoulders form a straight line. Hold briefly, then slowly lower your hips back down to the starting position.

**Quadriceps**

*Quad Sets*

Sit with leg out straight in front of you. Place small towel roll under knee. Tighten the muscles around your knee and press the towel into the mat, straightening your knee and lifting your heel off of the table. Hold for 5 seconds, then relax.

*Short Arc Quads*

Sit with leg out straight in front of you. Place a large rolled-up towel or object under your knee and slowly straighten your knee as your raise up your foot.

*Straight Leg Raise*

Lie comfortably on your back with one leg bent and the other leg straight. Flex the quadriceps in your straight leg maintain the knee in an extended position. Slowly raise the leg to the same angle as your bent knee. Hold briefly, then slowly lower the leg keeping your knee straight.

*Seated Knee Extension*

Sit in a firm chair or on a stable surface with your back straight and your legs at a 90º angle in front of you. Slowly straighten the knee until it is parallel to the floor. Hold briefly then lower slowly.

*Knee Extension on Machine*

Start with the knees in a flexed position. Push up with equal force with both legs until knees are straight. Come back down in a slow controlled movement.

*Terminal Knee Extension on Leg Press Machine*

Place feet up onto plate positioning them so that your knees are directly over ankles and knees are bent to about 15 degrees. Push plate forward until knees are straight. Slowly return to previous starting position.

*Leg Press Progressing Depth and Weight*

Place feet up onto plate positioning them so that your knees are directly over ankles and knees are bent to 90 degrees (or specific to patient progression). Push plate forward until knees are straight. Slowly return to previous position.

*Wall Squats with Yoga Ball Behind Back*

Start by standing up and leaning your low back up against an exercise ball on a wall. Your feet should be spread apart about shoulder width apart. Next, slowly bend your knees and lower your buttocks towards the floor. Knees should bend in line with the 2nd toe and not pass the front of the foot.

*Squat with Resistance Band*

While standing in a squatted position and holding an elastic band, extend your knees up to a standing position. Lower back down and repeat.

*Isometric Wall Squats*

Lean against wall with feet at least 2 feet from wall and shoulder width apart. Slide hips toward floor to sitting position, keeping your knees in line with your ankles. Hold 30 seconds; raise up; repeat.

*Squat while Holding Yoga Ball*

Hold a stability ball close to your chest. Stand with your feet about shoulder width apart. Hug the ball and go down into a squat. Do not allow your knees to pass your toes as you go into the squat position.

*Squat with Ball Between Knees*

Stand with ball placed between your knees. Squeeze ball and preform a squat, then stand back up again.

**Hamstrings**

*Standing Knee Bend*

Find a clear, smooth wall. Stand up straight bracing yourself against the wall. Bend one knee straight back towards your buttocks. Keep the leg straight behind you, do not let the leg rotate to the left or right. Hold briefly, then lower the leg back to the floor

*Hamstring Stool Pulls*

Sit up tall, dig your heels into the ground, and use full range of motion at your knee to move yourself forward.

*Hamstring Curls on Machine*

Seated: Start with your legs straight out in front of you, the backs of your lower legs on the padding. Using your hamstrings, pull the pad down towards the floor and return to start in one fluid motion.

Prone: Start with your knees straight with the padding resting on the back of your lower legs. Using your hamstrings, pull the pad up towards your buttocks and return to the starting position.

*Single Leg Romanian Deal Lift (RDL)*

1) Begin in tall standing position with a slight bend in both knees. 2) Engage your core by lightly bringing belly button closer to the spine to maintain neutral lumbar spine. 3) Begin to hinge at the hips while you maintain a neutral spine. The first movement should be backwards as if someone were pulling your weight/hips backwards. 4) Once you feel stretch in the hamstrings, begin to drive through your heels to bring your hips forward and shoulders back to starting position. 5) If you are using weight, keep the weight close to your body. Your shins should stay vertical and the knees should maintain the same amount of bend. 6) It is important to not allow the lumbar spine to hyperextend or flex, it should maintain neutral position!

**Function & Neuromuscular**

*Mini Wall Squats*

Stand with your back against the wall, with your feet shoulder width apart. Slowly lower your hips towards the ground. Keep your back as straight as possible and keep your knees under you such that you can always see your toes. Squat with your knees bent slightly, hold briefly, then return to a standing position.

*Sit-to-Sand from Chair*

Find a firm chair and stand directly in front of it. Stand straight up with your feet shoulder width apart. Slowly lower your hips towards the ground as if you are sitting down. Keep your back as straight as possible and keeps your knees under you such that you can always see your toes. Lower yourself all the way into the chair. Immediately rise again, slowly returning to a standing position.

*Staggered Sit-to-Stand from Chair*

Find a firm chair and stand directly in front of it. Stand straight up with one leg directly in front of you. Slowly lower your hips towards the seat as if you are sitting down, with most of your weight on the leg that is beneath you. Lower yourself all the way into the chair. Immediately rise again, slowly returning to a standing position.

*Marching Balance*

Stand with upright posture and abdominals contracted, slowly bend and straighten hip as if you were marching. Repeat as directed.

*Heel Tap Step Down*

Start with both feet on top of a step/box. Next, slowly lower the unaffected leg down off the side of the step/box to lightly touch the heel to the floor. Then return to the original position with both feet on the step/box. Maintain proper knee alignment: Knee in line with the 2nd toe and not passing in front of the toes.

*Step Up and Down (Anterior/Posterior/Lateral)*

Anterior/Posterior: Stand in front of a box. Step up as shown driving the body up with the leg on the box. Keep the body still by using the core. Stance-side foot and knee should remain pointed straight ahead. Step down in either the anterior or posterior direction.

Lateral: While standing next to a box or raised surface, step up and to the side on to the surface. Both feet should touch the raised surface. Then step down and onto the floor towards the same side that you started from.

*Lunge*

Start by standing with feet shoulder-width-apart. Next, take a step forward and allow your front knee to bend. Your back knee may bend as well. Then, return to original position, or you may walk and take a step forward and repeat with the other leg. Keep your pelvis level and straight the entire time. Your front knee should bend in line with the 2nd toe and not pass the front of the foot.

*Lunge while Holding Yoga Ball*

While in a standing position and holding a ball against your chest, step forward and bend your knees as you hold the ball forward and away from your chest. Next return to original standing position.

*Lunge onto Foam Pad*

Start by standing with feet shoulder-width-apart. Next, take a step forward onto the foam pad. Your back knee may bend as well. Then, return to original position. Keep your pelvis level and straight the entire time. Your front knee should bend in line with the 2nd toe and not pass the front of the foot.

*Incomplete Circles on Stable Surface*

Stand with feet hip width apart, then place your weight through one standing leg. Place a cloth or plastic bag underneath the circling leg. Using this leg, make clockwise and counterclockwise incomplete circles. Perform on left and right leg.

*Incomplete Circles with Yoga Ball*

Stand with feet hip width apart, then place your weight through one standing leg. Hold the yoga ball in front of you. Place a cloth or plastic bag underneath the circling leg. Using this leg, make clockwise and counterclockwise incomplete circles. Perform on left and right leg.

*Incomplete Circles on BOSU Ball*

Stand with feet hip width apart, then put one leg on the BOSU ball. Place your weight through the leg that is on the BOSU ball. Place a cloth or plastic bag underneath the circling leg. Using this leg, make clockwise and counterclockwise incomplete circles. Perform on left and right leg.

*Pelvic Tilt*

Lie on your back with your knees bent. Next, arch your low back and then flatten it repeatedly. Your pelvis should tilt forward and back during the movement. Move through a comfortable range of motion.

*Supine Bracing - Feet Flat*

Lie on your back with your knees bent. Tighten your stomach muscles are you draw your navel down towards the floor.

*Supine Bracing – Legs 90/90*

While lying on your back with your knees bent, raise up both feet. Use your stomach muscles to keep your spine from moving.

*Plank*

Hold a plank position in full elbow extension position with your legs spread slightly apart as shown. Do not let your back arch down.

*Dynamic Single Leg Squat*

Start in a single leg balance position. Begin to do a single leg squat with the balancing leg and at the same time the other leg will then reach back and across the midline and touch the toe to the ground. While leg is extended, make circles in either the clockwise or counter clockwise direction.

*Balance* a*nd Reach (Posterior/Anterior/Lateral)*

Stand next to a sturdy surface. Position 1: While standing on one leg, reach forward as far as you can with opposite leg while maintaining your balance. Keep the knee of the balancing leg soft or bent slightly. The goal is only to tap the foot, do NOT shift your weight on to the foot. Position 2: Reach to the side while maintaining your balance. Position 3: Reach back while maintaining your balance.
